# Targeting Affective Mood Disorders With Ketamine to Prevent Chronic Postsurgical Pain

**DOI:** 10.3389/fpain.2022.872696

**Published:** 2022-06-27

**Authors:** Dianna E. Willis, Peter A. Goldstein

**Affiliations:** ^1^Burke Neurological Institute, White Plains, NY, United States; ^2^Feil Family Brain and Mind Institute, Weill Cornell Medicine, New York, NY, United States; ^3^Department of Anesthesiology, Weill Cornell Medicine, New York, NY, United States; ^4^Department of Medicine, Weill Cornell Medicine, New York, NY, United States

**Keywords:** pain, dissociation, ketamine, HCN channel, oceanic boundlessness, out-of-body experience

## Abstract

The phencyclidine-derivative ketamine [2-(2-chlorophenyl)-2-(methylamino)cyclohexan-1-one] was added to the World Health Organization's Model List of Essential Medicines in 1985 and is also on the Model List of Essential Medicines for Children due to its efficacy and safety as an intravenous anesthetic. In sub-anesthetic doses, ketamine is an effective analgesic for the treatment of acute pain (such as may occur in the perioperative setting). Additionally, ketamine may have efficacy in relieving some forms of chronic pain. In 2019, Janssen Pharmaceuticals received regulatory-approval in both the United States and Europe for use of the S-enantiomer of ketamine in adults living with treatment-resistant major depressive disorder. Pre-existing anxiety/depression and the severity of postoperative pain are risk factors for development of chronic postsurgical pain. An important question is whether short-term administration of ketamine can prevent the conversion of acute postsurgical pain to chronic postsurgical pain. Here, we have reviewed ketamine's effects on the biopsychological processes underlying pain perception and affective mood disorders, focusing on non-NMDA receptor-mediated effects, with an emphasis on results from human trials where available.

## Introduction

**Figure 1:**
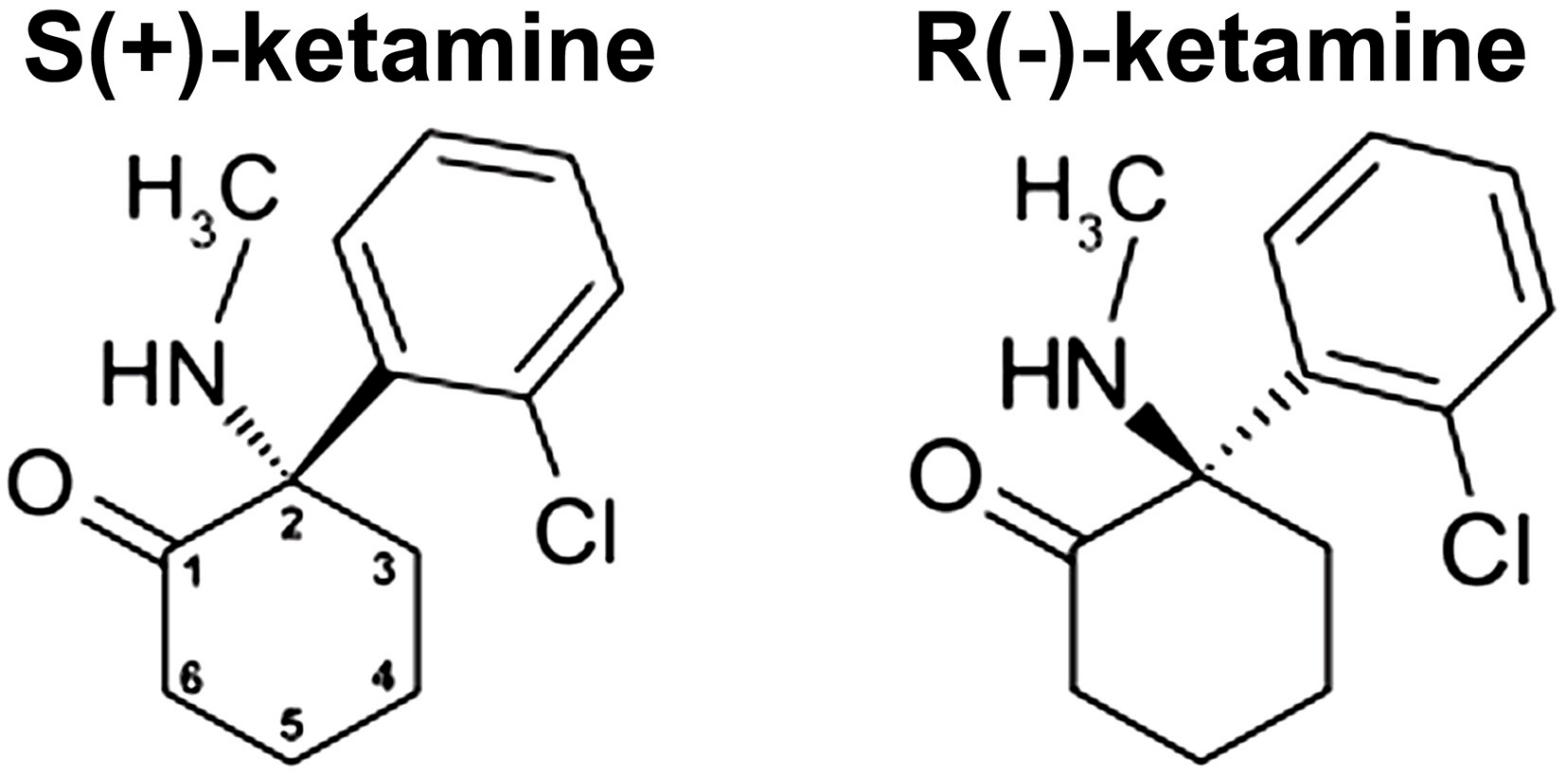
Ketamine structure. Ketamine [2-(2-chlorophenyl)-2-(methylamino)cyclohexan-1-one] contains a chiral center at C-2 of the cyclohexanone ring (numbered as shown), which gives rise to the two stereoisomers shown.

The phencyclidine-derivative ketamine [2-(2-chlorophenyl)-2-(methylamino)cyclohexan-1-one] was added to the World Health Organization's Model List of Essential Medicines in 1985 and is also on the Model List of Essential Medicines for Children due to its efficacy and safety as an intravenous anesthetic. In sub-anesthetic doses, ketamine is an effective analgesic for the treatment of acute pain (such as may occur in the perioperative setting) (1–3). Additionally, ketamine may have efficacy in relieving some forms of chronic pain (4, 5), including neuropathic pain (6). Not surprisingly, in those clinical studies of ketamine (*vs*. placebo) for the treatment of chronic non-cancer pain, there was a significant increase in the incidence of “psychedelic” side-effects in the ketamine group [risk ratio (95% confidence interval): 5.35; (2.64, 10.81)] (6). The relevance of this observation will be made clear below. The molecular structure contains a chiral center at C-2 of the cyclohexanone ring, thereby giving rise to R(-) and S(+) stereoisomers (Figure 1). In 2019, Janssen Pharmaceuticals received regulatory-approval in both the United States (7) and Europe (8) for use of the S-enantiomer of ketamine in adults living with treatment-resistant major depressive disorder. Pre-existing anxiety/depression and the severity of postoperative pain are risk factors for development of chronic postsurgical pain. An important question is whether short-term administration of ketamine can prevent the conversion of acute postsurgical pain to chronic postsurgical pain.

**Figure 2:**
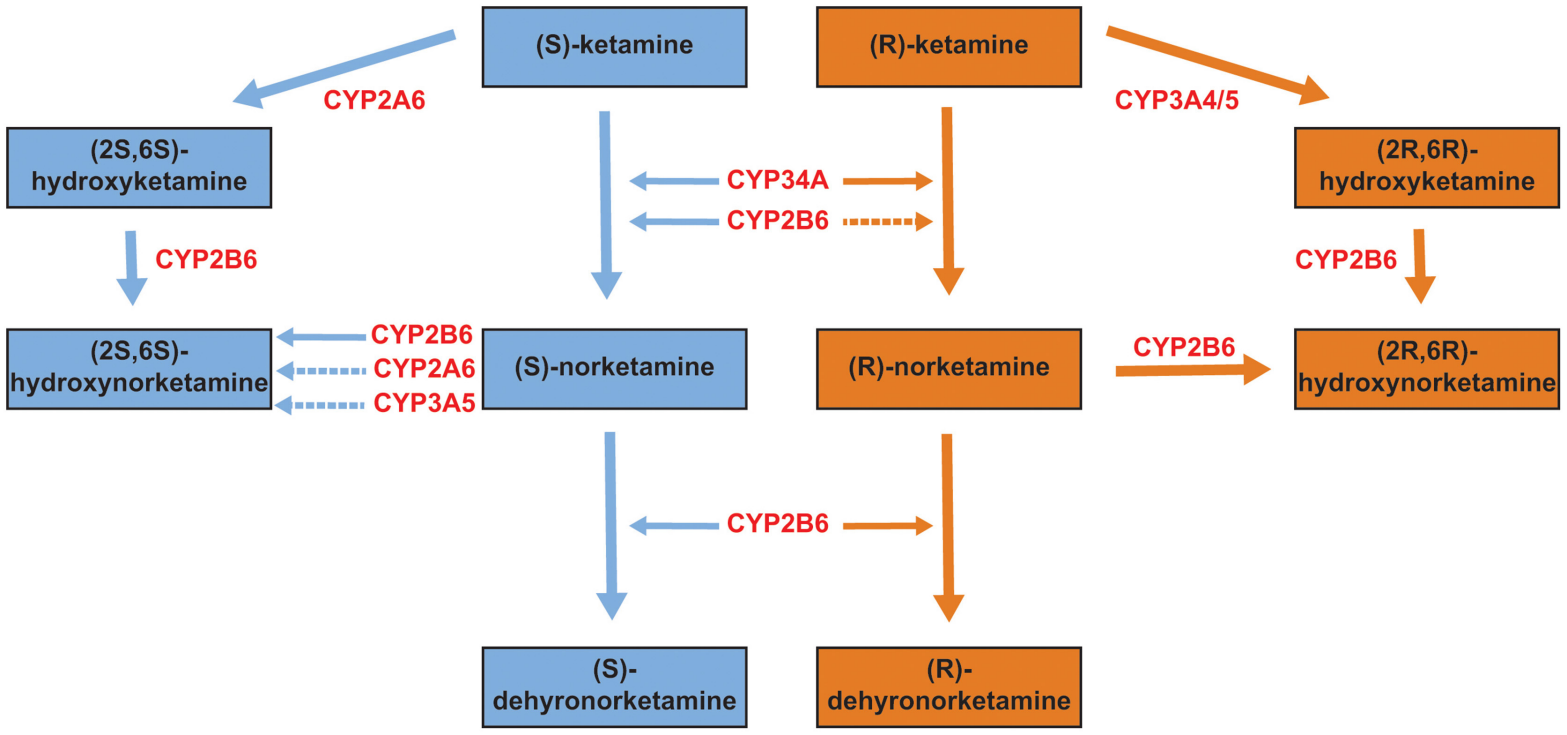
Primary metabolites of ketamine. Ketamine exists in (S) and (R) configurations (see Figure 1). Each is metabolized to several primary metabolites, with the relevant hepatic enzymatic pathways shown in red (primary pathways are indicated by solid arrows, secondary/minor pathways by dotted arrows). Data from (9, 10).

Ketamine was first synthesized in the early 1960s as an alternative to phencyclidine (PCP), which had been approved by the United States Food and Drug Administration as an anesthetic, but which had side effects that limited its usefulness. Unless otherwise specified, ketamine, when used clinically, is administered as the racemate, (R,S)-ketamine. In the predominant metabolic pathway, (R,S)-ketamine is initially metabolized to norketamine [(R,S)-norketamine]. (R,S)-Norketamine in turn can be metabolized in a stereoselective manner to form both (S) and (R) dehydronorketamines (DHNKs) and hydroxynorketamines (HNKs) (Figure 2). Hydroxylation at the six position of norketamine results in (2R,6S;2S,6S)-hydroxynorketamine while hydroxylation in the four position (by CYP2B6 or CYP2A6) results in the 4-hydroxy isomers, (2R,4R;2S,4S)-hydroxynorketamine and (2R,4S;2S,4R)-hydroxynorketamine. Lastly, hydroxylation of norketamine at the five position by CYP2B6 produces (2R,5S;2S,5R)-hydroxynorketamine and (2R,5R;2S,5S)-hydroxynorketamine. (R,S)-dehydroxynorketamine can result either from direct dehydrogenation from norketamine or dehydration from either diastereomer of the 5-hydroxynorketamines. There are additional minor metabolic pathways that result in low abundance metabolites, which include: hydroxyphenyl-ketamine, hydroxyphenyl-norketamine, 4-hydroxyketamine, (2R,6S;2S,6R)-hydroxynorketamine, (2R,6S;2S,6R)-hydroxyketamine, and (2R,6R;2S,6S)-hydroxyketamine (9). With respect to the individual enantiomers, (R)-ketamine selectively forms (2R,6R)-hydroxynorketamine while (S)-ketamine selectively forms (2S,6S)-hydroxynorketamine (9–11).

The dose of ketamine resulting in unresponsiveness in humans is ~ 2 mg/kg iv, which produces plasma concentrations of 1,500–1,800 ng/ml (~6.3–~7.6 μM) and 2,000 ng/ml (~8.4 μM) in adults (12–14) and children (13), respectively; consciousness reappears when the concentration falls below 1,060 ng/ml (4.5 μM) (12, 14). In healthy, young (36 ± 3 yr, *n* = 5) human volunteers, (R,S)-ketamine (50 mg/min), (S)-ketamine (25 mg/min), or (R)- ketamine (75 mg/min) was administered over 5–7-min by continuous infusion; the infusion was discontinued after 5 min (or when *no* further changes were observed in the EEG over a 30–60 sec interval). The total doses (mean ± SD) of (R,S)-ketamine, (S)-ketamine, and (R)-ketamine were 275 ± 25 mg, 140 ± 21 mg, and 429 ± 37 mg, respectively. Serum ketamine concentrations associated with regaining consciousness and orientation were consistent with an (S):(R) isomer potency ratio of 4:1 [Table 1; (15)]. With regards to psychomotor function impairment, the (S):(R) potency ratio varied from 3:1 to 5:1. After comparable degrees of CNS depression as measured by electroencephalography, (S)-ketamine was associated with a more rapid recovery of psychomotor skills than (R,S)-ketamine.

**Table 1:** Time to behavioral recovery and corresponding serum ketamine concentrations in healthy human volunteers.

**Orientation variable**	**(R,S)-ketamine**		**S(+)-ketamine**		**R(-)-ketamine**	
	**Time (min)**	**μg/ml**	**Time (min)**	**μg/ml**	**Time (min)**	**μg/ml**
		**[μM]**		**[μM]**		**[μM]**
Opened eyes	11 ± 3	2.6 ± 0.7	8 ± 1	1.2 ± 0.3^*^	7 ± 2	5.2 ± 0.8^*^
		[10.9 ± 2.9]		[5.0 ± 1.3]		[21.9 ± 3.4]
Squeezed hands	22 ± 8	1.6 ± 0.5	12 ± 3	1.0 ± 0.3	9 ± 2^*^	4.1 ± 0.9^*^
		[6.7 ± 2.1]		[4.2 ± 1.3]		[17.2 ± 3.8]
Oriented to person	33 ± 11	1.2 ± 0.3	14 ± 2^*^	0.9 ± 0.2	10 ± 2^*^	3.9 ± 0.8^*^
		[5.0 ± 1.3]		[3.8 ± 0.8]		[16.4 ± 3.4]
Oriented to person, place, time	45 ± 10	1.0 ± 0.1	21 ± 2^*^	0.7 ± 0.2^*^	18 ± 3^*^	2.7 ± 0.5^*^
		[4.2 ± 0.4]		[2.9 ± 0.8]		[11.4. + 2.1]
**Psychomotor test**
Analogue scales	161 ± 21	0.4 ± 0.1	150 ± 22	0.2 ± 0.03^*^	123 ± 19	0.7 +0.2^*^
		[1.7 ± 0.4]		[0.8 ± 0.1]		[2.9 ± 0.8]
Trigger test	164 ± 17	0.4 ± 0.1	87 ± 13^*^	0.3 ± 0.1^*^	65 ± 9^*^	1.1 ± 0.3^*^
		[1.7 ± 0.4]		[1.3 ± 0.4]		[4.6 ± 1.3]
Symbol–digits	178 ± 19	0.4 ± 0.1	122 ± 29	0.2 ± 0.1^*^	104 ± 19^*^	0.8 +0.3^*^
		[1.7 ± 0.4]		[0.8 ± 0.4]		[3.4 ± 1.3]
Time distortion	118 ± 24	0.5 ± 0.2	74 ± 12^*^	0.3 ± 0.1^*^	57 ± 20^*^	1.2 ± 0.8
		[1.7 ± 2.1]		[1.3 ± 0.4]		[5.0 ± 3.4]

* *P < 0.05 compared to racemate. Data (n = 5 subjects (male); mean ± SD) from White et al. (15)*.

In contrast, the ketamine concentration (and corresponding dose) required to provide acute pain relief is markedly less. In healthy adult volunteers, a single bolus dose administered intravenously of either 0.125 or 0.25 mg/kg produces peak plasma concentrations of ~60 ng/ml [~0.25 μM] and 175 ng/ml [0.74 μM], respectively (16), while a single dose of 0.5 mg/kg administered intramuscularly results in an average peak concentration of 240 ± 50 ng/ml [1.0 ± 0.2 μM] (17). Regardless of the route of administration, marked analgesia is seen at concentrations < 250 ng/ml (< 1.1 μM) (16–18). It is worth noting that the concentration of ketamine associated with analgesia is 2–3× of that required for its antidepressant effects in patients with treatment-resistant major depression (following 0.5 mg/kg (R,S)-ketamine infused over 40 min, average peak plasma concentration was 128 ± 44 ng/ml [0.54 + 0.19 μM] (19), which is comparable to population model estimates of 72.5–96.6 ng/ml [0.31–0.41 μM] for the treatment-approved intranasal dose of 56 mg (S)-ketamine (20); see also Table 2).

**Table 2:** Average plasma concentrations of ketamine and selected metabolites in patients with treatment resistant depression following 40 min infusion of 0.5 mg/kg (R,S)-ketamine.

**Time (min)**	**Concentration (ng/ml, [*μM*])**				
	**40**	**80**	**110**	**230**	**1,440**
**Metabolite**					
**Bipolar depression**					
(R,S)-ketamine	177.23 ± 53.8	83.21 ± 28.17	60.02 ± 25.01	27.63 ± 14.52	9.19 ± 10.92
	[0.75 ± 0.23]	[0.35 ± 0.12]	[0.25 + 0.11]	[0.12 + 0.06]	[0.04 ± 0.05]
(R,S)-norketamine	63 ± 24.82	69.96 ± 19.98	63.35 ± 20.55	43.49 ± 16.88	14.36 ± 9.27
	[0.27 ± 0.10]	[0.29 ± 0.08]	[0.27 ± 0.09]	[0.18 ± 0.07]	[0.06 ± 0.04]
(R,S)-dehydroxynorketamine	28.07 ± 18.72	48.07 ± 26.43	50.5 ± 27.44	43.08 ± 23.76	16.87 ± 13.51
	[0.12 ± 0.08]	[0.20 ± 0.11]	[0.21 ± 0.12]	[0.18 ± 0.1]	[0.07 ± 0.06]
**Major depressive disorder**					
(R,S)-ketamine	204.13 ± 101.46	93.5 ± 31.06	65.03 ±23.17	33.86 ± 19.04	BLQ
	[0.86 ± 0.43]	[0.39 ± 0.13]	[0.27 ± 0.1]	[0.14 ± 0.08]	
(R,S)-norketamine	55.52 ± 33.87	73.54 ± 31.86	62.74 ± 26.78	46 ± 22.97	12.39 ± 8.47
	[0.23 ± 0.14]	[0.31 ± 0.13]	[0.26 ± 0.11]	[0.19 ± 0.1]	[0.05 ± 0.04]
(R,S)-dehydroxynorketamine	7.52 ± 4.8	12.02 ± 6.19	13.27 ± 6.92	10.17 ± 6.65	BLQ
	[0.03 ± 0.02]	[0.05 ± 0.03]	[0.06 ± 0.03]	[0.04 ± 0.03]	

*BLQ, below limits of quantitation. Data from Zarate et al. (21). Patients with treatment resistant depression included those with major depressive disorder (MDD; n = 45) and bipolar depression (BD; n = 22)*.

Hyperpolarization-activated cyclic nucleotide-regulated type 1 (HCN1) ion channels have recently gained attention as highly relevant molecular targets for a wide range of general anesthetics (22), including ketamine (23, 24). HCN channels, of which there are four isoforms (HCN1-4), belong to the K_V_ channel superfamily, and are responsible for the generating the “pacemaker” current I_h_ (25–29). The channels assemble as homo- and hetero-tetramers [with only HCN2-3 disfavored (30)] and are present throughout the nervous system (31, 32).

In HEK293 cells transiently transfected with mouse (m)HCN1, (R,S)-ketamine and S-ketamine significantly shifted the V_1/2_ (i.e., the voltage required for half-maximal current activation) to more hyperpolarized potentials (as measured by the ΔV_1/2_) with EC_50_s of 8.2 ± 1.2 μM and 4.1 ± 1.2 μM, respectively, with corresponding maximal ΔV_1/2_ values of −11.9 ± 0.7 mV and −14.5 ± 0.9 mV (23). Even at their EC_50_s, both (R,S)-ketamine and (S)-ketamine left-shifted the V_1/2_ by ~4–5 mV, which would limit membrane repolarization following hyperpolarization, thereby preventing a neuron from reaching action potential threshold. Of note, HCN1 and HCN2 will freely co-assemble given the opportunity *in vitro* (30, 33) and *in vivo* (34), and the effect of ketamine on mHCN1-mHCN2 heteromers was comparable to that on mHCN1 homomers (23). These observations raise the intriguing possibility that modulation of HCN1 channel function contributes to the efficacy of ketamine as an antidepressant [for reviews see (35, 36)], which in turn may be a critical determinant in whether short-term administration of ketamine can prevent the conversion of acute postsurgical pain to chronic postsurgical pain.

## Use of Ketamine in Pain States

### Acute Pain

Ketamine gained Food and Drug Administration approval in 1970 for the clinical use as an anesthetic in humans. Since that time, ketamine has been used for purposes beyond its role as an anesthetic. One of the primary uses has been in the perioperative setting, where it can serve as both part of an anesthetic “cocktail” and as a means of managing acute pain. Its use at subanesthetic doses for acute pain management is so prevalent that consensus guidelines have recently been developed (1). Ketamine's likely mechanism of action in acute pain is *via* its antagonism of the *N*-methyl-D-aspartate (NMDA) receptor, where it is a non-competitive antagonist with an IC_50_ of 1.6 μM (Table 3).

**Table 3:** Ketamine and putative biological targets.

**Site**	**Value (μM)**	**Type**	**Action**	**Species**	**References**
**Acetylcholine receptors**					
M_1_	45	K_i_	ND	Human	(37)
α_2_β_2_	92	IC_50_	Antagonist	Human	(38)
α_2_β_4_	29	IC_50_	Antagonist	Human	(38)
α_3_β_2_	50	IC_50_	Antagonist	Human	(38)
α_3_β_4_	9.5	IC_50_	Antagonist	Human	(38)
α_4_β_2_	72	IC_50_	Antagonist	Human	(38)
α_4_β_4_	18	IC_50_	Antagonist	Human	(38)
α_7_	3.1	IC_50_	Antagonist	Rat	(39)
**Dopamine receptors**					
D_2_	0.5	K_i_	Agonist	Human	(40)
	>10	K_i_	ND		(41–43)
**Estrogen receptors**					
ERα	0.34	K_i_	ND	Human	(44)
**Glutamate receptors**					
NMDA	0.25–0.66	K_i_	Antagonist	Human	(41, 45)
	1.6	IC_50_	Antagonist	Rat	(46)
**Opiate receptors**					
MOR	42	K_i_	Antagonist	Human	(47)
MOR_2_	12.1	K_i_	Antagonist	Human	(48)
KOR	28	K_i_	Antagonist	Human	(47)
	25	K_i_	Agonist		(49)
**Sigma receptors**					
σ_2_	26	K_i_	ND	Rat	(50)
**Transporters**					
NET	82–291	IC_50_	Inhibitor	Human	(51, 52)
DAT	63	K_i_	Inhibitor	Rat	(51)
**Ion channels**					
Calcium channels					
α1G (Ca_V_3.1)	1,200	IC_50_	inhibitor	Rat	(53)
**Potassium channels**					
BK	4.1–230	IC_50_	Inhibitor	Rat (GH3 cells)	(54)
HCN1	8–16	EC_50_	Inhibitor	Mouse	(23)
K_ATP_	62.9	K_i_	Inhibitor	Rat	(55)
SK2	470	K_D_	Inhibitor	Rat	(56)
**Sodium channels**					
Skeletal (hSkM1; Na_V_1.4)	(S)-ketamine: 191	IC_50_	Inhibitor	Human	(57)
	(R)-ketamine: 387				
Neuronal (brain IIa; Na_V_1.2)	(S)-ketamine: 529	IC_50_	Inhibitor	Rat	(57)
	(R)-ketamine: 648				

The noncompetitive antagonism of NMDA reduces glutamate release, and the resulting reduction in this excitatory neurotransmitter attenuates pain sensation transmission [for review, see (58)]. While this is likely the predominant mode of action underlying its effectiveness in acute pain reduction, ketamine does not exclusively act upon the NMDA receptor. It has known activity on μ-opioid receptors, acetylcholine receptors, and hyperpolarization activated cyclic nucleotide-regulated channels, among others (Table 3). These targets may also be critical mediators of acute pain reduction. Numerous studies have demonstrated that the use of perioperative ketamine reduces postsurgical pain scores and opioid consumption (59–62). Based on meta-analysis of available studies, the use of ketamine in the perioperative setting for the reduction of moderate to severe acute pain rises to Grade B level, such that there is moderate to high certainty that there will be moderate to substantial benefit (1). This has been supported by a recent meta-analysis and systematic review of ketamine use following breast surgery. Here, the authors found that ketamine reduced wound pain during the first 6 and 24 h after surgery, and also decreased opioid consumption during the first 24 h (63). Taken together, there is considerable evidence of the efficacy of using ketamine, particularly in the perioperative setting, for the treatment of acute pain.

### Chronic Pain

While the evidence for the use of ketamine in acute pain, and the proposed mechanisms of action, appear clear, there is considerably more debate about the efficacy of ketamine use in chronic pain. Much of this uncertainty likely revolves around the variability in patient condition, dosing regimen, mode of administration, and issues surrounding monitoring. Given the preponderance of chronic pain, and its impact on quality of life and lost productivity, clarification surrounding these points is critical. This is especially true given the problems associated with long term opioid use and the potential for abuse.

Abundant evidence for its potential use in treating chronic pain come from preclinical animal studies. Many chronic pain states, especially those that are neuropathic in nature, demonstrate elements of central sensitization. The prevailing theory for ketamine's chronic pain effects are by attenuating the effects of central sensitization *via* its antagonism of NMDA receptor activity; however, there is considerable debate about the mechanism(s) of action of ketamine and its metabolites for their ability to attenuate chronic pain. In a recent study, multiple intraperitoneal injections of the ketamine metabolite (2R,6R)-hydroxynorketamine [(2R,6R)-HNK] results in reduction in mechanical allodynia for several days in multiple chronic pain and postoperative pain models. Interestingly, ketamine itself did not produce this sustained pain relief (64). An early paper comparing ketamine, its active metabolite norketamine, and selective inhibitors of the NR2 subunit of the NMDA receptor, found that while these selective inhibitors could provide chronic pain relief, they were significantly less potent than ketamine (65). This points to non-NMDA-mediated effects contributing to the attenuation of chronic pain. Taken together, the preclinical animal studies support the efficacy of ketamine to provide relief of chronic pain; however, they further highlight the complexity of the proposed mechanism(s). Once again, non-NMDA ketamine targets, such as HCN1 channels, cholinergic, aminergic, and opioid receptors, as well as downstream modulation of signaling pathways such brain derived neurotrophic factor (BDNF) and others, may be critical components of these effects on chronic pain. In contrast to other analgesics, the effects of ketamine appear to be uncoupled from its pharmacokinetics, with effects persisting at points where plasma concentrations of ketamine and its metabolites are low or undetectable (66). The mechanism(s) accounting for these persistent effects are not fully understood; however, they suggest that connectivity changes in specific brain networks may contribute to the long-lasting pain relief. Putative brain networks promoting these effects are discussed below. Further preclinical studies will be needed to untangle the complex role of ketamine in chronic pain.

Sufficient preclinical evidence supports the use of ketamine in chronic pain relief. Unfortunately, many human studies have been poorly controlled and underpowered, resulting in a lack of clarity regarding human efficacy for this condition. In randomized controlled, double-blind studies, ketamine was found to provide better pain relief than placebo in subjects with mixed neuropathic pain; however, this improvement was often short in duration and inconsistent across trials (67–69). Similar trials in subjects with traumatic spinal cord injury, phantom limb pain, or postherpetic neuralgia show similar improvements, though often not demonstrating long-lived effects (70–72). Based on an analysis of these and other studies, recent consensus guidelines on the use of intravenous ketamine for chronic pain have been developed (4). They concluded that the best evidence for efficacy, rising to the level of Grade B evidence, is for the treatment of complex regional pain syndrome. Evidence for other pain conditions is weak with low certainty, and additional well-designed trials are needed. To date, the use of ketamine in chronic pain management is limited by the apparent need for frequent administration, the need for long duration of administration, the disparate results of clinical trials, and the failure to identify treatment approaches that may be curative (66, 73).

## Transition From Acute to Chronic Pain and the Development of Chronic Postsurgical Pain

An intriguing question is to what extent ketamine may be able to prevent the transition from acute to chronic pain. Determining the potential for ketamine use in this regard is predicated on a better understanding of how this transition occurs. Measurements of the prevalence of chronic pain vary widely depending on the methods used for calculation and the means by which pain is classified as chronic; however, reasonable estimates may indicate that 20% of the general population suffer from some form of chronic pain (74). In order to better study and address means of treatment, the International Association for the Study of Pain (IASP) has defined chronic pain as pain that lasts or recurs for longer than 3 months (75). The development of chronic pain, and the transition from an acute pain state into a chronic state, is multimodal, encompassing physiological, neurobiological, psychological, and psychosocial factors. One of the most common instances of the chronification of acute pain is chronic postsurgical pain (CPSP). Nearly 10% of all surgical procedures result in CPSP sufficient to cause functional impairment (76). Based on this estimate, more than 30 million people annually may be at risk of developing CPSP (77). While this clearly has a significant impact on quality of life and loss of productivity, it is also a significant driver of the current opioid crisis (78). As in other forms of chronic pain, CPSP can have alternative definitions. The current consensus definition, as evidenced by its inclusion in International Classification of Disease (79), is:

*Chronic postsurgical pain is chronic pain developing or increasing in intensity after a surgical procedure and persisting beyond the healing process, (i.e., at least 3 months after surgery). The pain is either localized to the surgical field, projected to the innervation territory of a nerve situated in this area, or referred to a dermatome (after surgery/injury to deep somatic or visceral tissues). Other causes of pain including infection, malignancy etc. need to be excluded as well as pain continuing from a pre-existing pain problem. Dependent on type of surgery, chronic postsurgical pain often may be neuropathic pain*.

As mentioned above, the transition from acute to chronic pain is driven by many elements. At the molecular level, animal models of chronic pain have demonstrated changes along the entire length of the pain circuit, from the peripheral sensory neurons to disinhibition in the dorsal horn of the spinal cord and higher-level processing in the brain (80, 81). In CPSP, the tissue damage caused during surgery initiates a cascade of events that can drive change in this circuit. Among these is the release of glutamate and the change in NMDA receptor composition and activation. While the exact mechanism(s) that result in CPSP are still not clear, it is likely due to peripheral and central sensitization initiated, at least in part, by these acute changes and by the degree and persistence of the postsurgical acute pain (82). In one study looking at pain following thoracic surgery, uncontrolled postoperative pain was the primary risk factor for developing CPSP (83).

The choice of anesthetic and analgesics used in the perioperative setting has been proposed to play a role in the development of CPSP. Curiously, the use of high-dose opioids for pain relief results in hyperalgesia, an increase in postoperative pain, and ultimately the use of higher amounts of opioids (82). Given that the degree and persistence of postsurgical pain has been linked to the development of CPSP, this suggest that postsurgical opioid use may in fact trigger this condition. As discussed above, studies have demonstrated that the use of perioperative ketamine reduces post-surgical pain scores and opioid consumption (59–62), pointing to the potential for ketamine to prevent the development of CPSP by reducing the use of opioids. In addition to reducing opioid hyperalgesia, preoperative ketamine use has been shown to inhibit dorsal horn sensitization and NMDA wind-up as well as activating inhibitory modulation (84, 85). Each of these are a viable putative mechanism for how ketamine might prevent the development of CPSP. The risk factors associated with the development of CPSP, beyond the degree of postsurgical pain discussed above, may point to other unexpected targets of ketamine's actions. Given the planned nature of surgical procedures, prevention may present an ideal means of combating CPSP, and the ability to identify those at highest risk is therefore essential.

## Risk Factors for the Development of Chronic Postsurgical Pain

Given the prevalence and ramifications of CPSP, significant effort has gone in to understanding the risk factors associated with its development. Several preoperative characteristics have been linked to an increased risk of CPSP across numerous surgical types. A recent systematic review and meta-analysis following breast and thoracic surgery identified some common characteristics (86). Among these was a younger age, an association between moderate to severe acute pain and CPSP, and an association between preoperative chronic pain and CPSP. These results seem to be broadly generalizable to other types of surgical procedures. For example, a recent prospective study identified similar risk factors for developing chronic pain following colorectal surgery (87). Sensitization of the pain pathways resulting from preoperative pain may lead to neuropathic pain and may contribute to the high incidence of CPSP in patients with high levels of preoperative pain (88). In addition to the degree of post- and preoperative pain, several psychological traits showed an association with CPSP. Among these are higher levels of anxiety, depression, and pain catastrophizing. This is consistent with prior systematic reviews that found significant associations between CPSP and depression, anxiety, catastrophizing, mental health, kinesiophobia (i.e., the fear of pain resulting from movement), and self-efficacy (89). Among these, state anxiety was the biggest predictor. State anxiety is anxiety in response to a specific dangerous or threatening situation, and in many cases may be associated with the planned surgery itself. State anxiety and pain catastrophizing have recently been the focus of CPSP interventional efforts (90). Among these approaches have been both pre- and post-operative behavioral therapies, which have shown some evidence for effectiveness at improving postoperative pain (91, 92). These results highlight that both chronic and acute psychological factors may be predictive of increased risk of developing CPSP. As such, targeting these risk factors *via* perioperative interventions, both psychological and pharmacological, may prevent the development of CPSP.

## Molecular Mechanisms Contributing to the Antidepressant Actions of Ketamine

### Ionotropic Glutamate Receptors

As discussed above, ketamine administration has clear antidepressant activity. The molecular mechanism(s) of this activity is a matter of intense interest, with arguments made for, and against, involvement of ionotropic glutamate receptors. In acutely prepared rat brain slices, ketamine, in a concentration-dependent manner, rapidly increased the slope of the field excitatory postsynaptic potential (fEPSP) at Schaffer collateral (SC)-CA1 synapses with an EC_50_ of 0.053 μM, and this was accompanied by a significant increase in the phosphorylation of the AMPA receptor GluA1 subunit (93). Preincubation of the slice with the protein kinase A (PKA) inhibitor H89 blocked both ketamine-induced potentiation of the fEPSP and GluA1 Ser^845^ phosphorylation. In the forced swim test (FST; in which a low immobility time is an indicator of an antidepressant-like response), a single intraperitoneal injection of ketamine (10 mg/kg) significantly reduced immobility in Sprague-Dawley rats. Compared with CA1 tissue from placebo treated rats, ketamine again increased GluA1 phosphorylation and abundance. In GluA1 S845A knock-in mice (wherein the serine to alanine substitution renders that site resistant to PKA phosphorylation), however, ketamine: 1) failed to enhance SC-CA1 fEPSPs in slices from GluA1 S845A mice (but strongly enhanced fEPSPs in slices from wild-type littermates), 2) failed to increase the membrane surface expression of GluA1 in GluA1 S845A mice, and 3) failed to reduced immobility of GluA1 S845A mice in the FST but significantly did so in wild-type mice. To test whether presynaptic modifications contributed to the observed synaptic potentiation, the effects of ketamine were examined using a conditional gene knock-out strategy wherein deletion of the GluN1 (NR1) subunit of the NMDA receptor was restricted to CA3 neurons (whose axons give rise to the Schaffer collaterals). *In vitro*, ketamine failed to potentiate the fEPSP in brain slices from CA3 GluN1-null mice (but did so in CA1 GluN1-null mice). *In vivo*, ketamine reduced the latency to feeding and duration of immobility in the FST in CA3 GluN1 null, but not CA1 GluN1-null, mice. Collectively, these observations strongly support a role for postsynaptic AMPA receptors and presynaptic NMDA receptors as necessary for the antidepressant activity of ketamine, and the importance of AMPA receptors as targets is in agreement with the demonstration that activation of AMPA receptors is required for both the acute and sustained antidepressant effects of (2R,6R)-hydroxynorketamine (94). The role of NMDA receptor blockade in this process, however, is complicated by other observations demonstrating that the anti-depressant effects of (R,S)-ketamine and its enantiomers appear to be independent of NMDA blockade [(11, 94); for review, see (95, 96)]. Regardless of the exact role of NMDA receptors *per se*, the question remains as to upstream regulation of ionotropic glutamate receptor function.

### Hyperpolarization-Activated Cyclic Nucleotide (HCN)-Regulated Ion Channels

HCN channels are expressed across the cell membrane, including at the axon terminal (97–103), where they regulate both inhibitory (99) and excitatory (101, 102) transmitter release. Zhang and colleagues examined whether HCN channels might contribute to the effects described above (93). Application of the pan-HCN isoform blocker ZD7288 occluded ketamine-induced SC-CA1 EPSC potentiation as well as increases in GluA1 phosphorylation and expression. Correspondingly, in HCN1 null mice, the fEPSP was not potentiated following application of ketamine. At the behavioral level, ketamine had no effect on either the sucrose preference or novelty-suppressed feeding test (both of which assess depression-like behaviors) in HCN1-null mice whereas both showed significant improvement in wild-type mice. These data demonstrate that HCN1 channels are necessary for the antidepressant activity of ketamine.

In order to discuss the role of ketamine-mediated inhibition of HCN channels in humans, we first need to consider the following question: Does the baseline state of awareness at the time of ketamine administration influence its antidepressant activity? This question was partly addressed in a prespecified analysis of the Prevention of Delirium and Complications Associated with Surgical Treatments (PODCAST) study (104), which was an international, multicenter, double-blind, randomized clinical trial that compared two doses of sub-anesthetic ketamine (0.5 mg/kg and 1 mg/ kg; low- and high-dose, respectively) with placebo for the prevention of postoperative delirium and pain in older adults (age > 60 years) undergoing major cardiac and non-cardiac surgery (105). Of critical importance, ketamine was administered *after* induction of general anesthesia (i.e., once loss of consciousness was achieved). Patients were screened for symptoms of depression at baseline using the Patient Health Questionnaire 8 (PHQ-8) scale and tested again on postoperative day (POD) 3 and ~POD 30. Patients were dichotomized into two groups—those with “symptoms suggestive of depression” and those “without symptoms of depression” based on a score of 10 or higher on a 0–24 PHQ-8 scale (106). The Consort flowchart of subjects indicates that 670 individuals completed the PHQ-8 questionnaire; of that number, 221 were allocated to the placebo group, 226 to the low-dose group, and 223 were allocated to the high-dose group, with 137, 130, and 128 subjects, respectively, completing the PHQ-8 on POD 30 for use in the final analysis. The proportion of subjects with symptoms suggestive of depression was not statistically different among the three groups (placebo−8.1%, low-dose−10.6%, and high-dose−9.9%). Surprisingly, neither dose of ketamine improved PHQ-8 scores at either time point. Thus, administration of ketamine following anesthetic-induced loss of consciousness does not improve symptoms of depression. Of course, *symptoms* of depression is not the same as a diagnosis of major depression, but a subanesthetic dose of (R,S)-ketamine does improve typical/melancholic and atypical symptoms of depression (as measured by the Montgomery-Asberg Depression Rating Scale (MADRS; for general depressive symptoms), MADRS5 (for typical/melancholic symptoms), and the Scale for Atypical Symptoms (SAS, for atypical symptoms) in patients with treatment-resistant major depressive disorder of bipolar depression (107).

**Figure 3:**
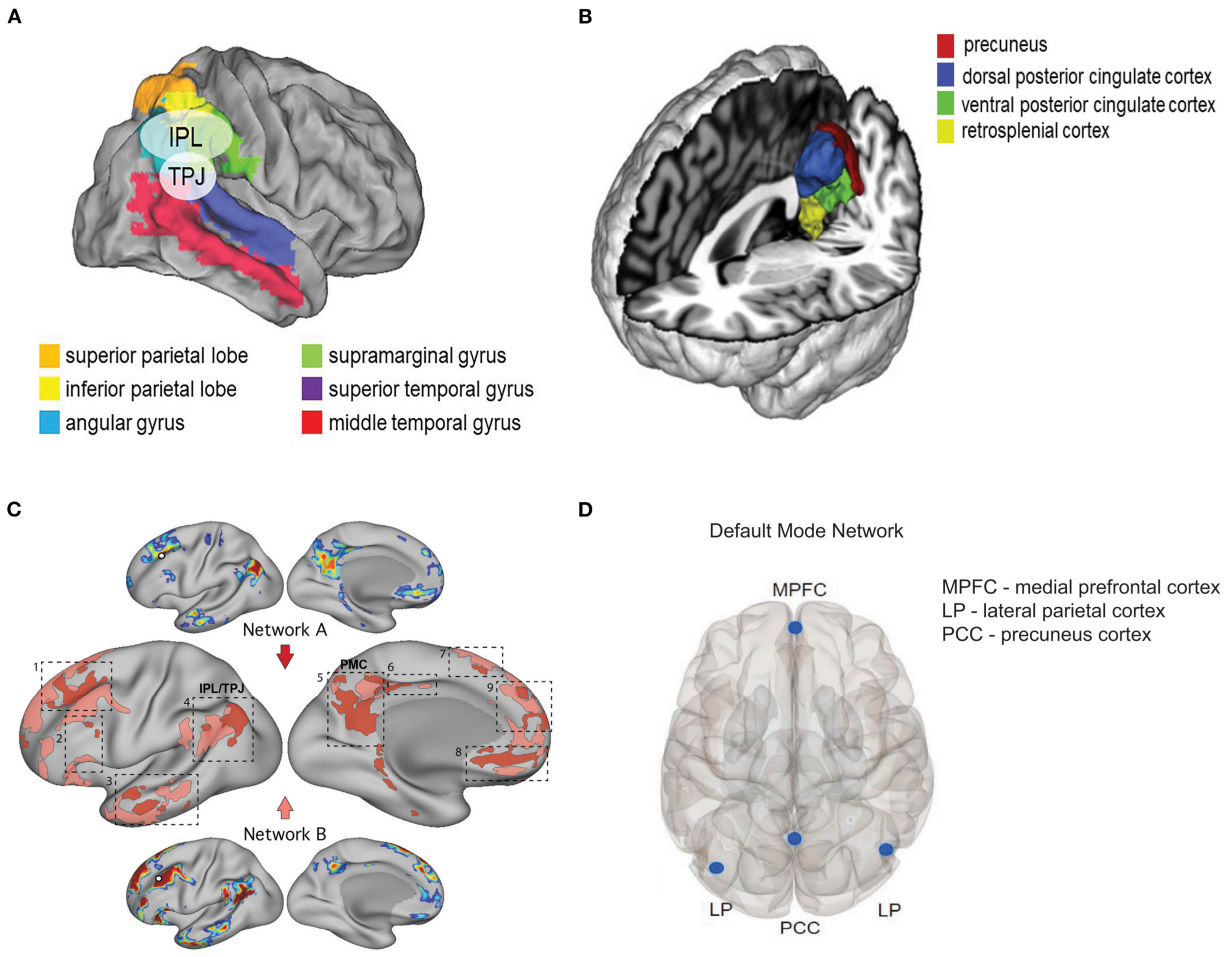
Neuroanatomical and functional networks relevant to ketamine's dissociate properties. **(A)** Map illustrating the relative anatomic position of the temporoparietal junction (TPJ) and the inferior parietal lobe (IPL). Maps are depicted on the flattened brain surface of the PALS atlas (109). Legend and image modified from Geng and Vossel (110) Figure 2 with permission under the terms of the Creative Commons Site License (https://creativecommons.org/licenses/by/4.0/). **(B)** Relevant structures that comprise the posterior medial cortex (PMC) as determined by whole-brain activation profiles. Image modified from Bzdok et al. (111) Figure 4 with permission. **(C)** Shown are two dissociated networks (A and B) near the default mode network (DMN) in a single subject. The dashed boxes highlight nine cortical zones where neighboring representations of the two networks were found, including: 1) dorsolateral prefrontal cortex (PFC), 2) inferior PFC, 3) lateral temporal cortex, 4) inferior parietal lobule (IPL) extending into the temporoparietal junction (TPJ), 5) posteromedial cortex (PMC), 6) midcingulate cortex, 7) dorsomedial PFC, 8) ventromedial PFC, and 9) anteromedial PFC. Note the proximity of zones 4 (TPJ/IPL) and 5 (PMC). Legend and image modified from Braga and Buckner (112) Figure 3 with permission under the terms of the Creative Commons Site License (https://creativecommons.org/licenses/by/4.0/). **(D)** Regions of interest that form the default mode network. Image modified from Krönke et al. (113) Figure 1 with permission.

Multiple factors could have led to a negative result, among which are the lack of a true diagnosis of major depression, confounding effects of general anesthetics, and insufficient sample size. Which brings us back to the original question: Might the state of awareness at time of administration matter? Subanesthetic doses of ketamine profoundly alter states of consciousness without producing loss of consciousness. In healthy adult volunteers (7 male, 8 female), subanesthetic ketamine (0.5 mg/kg infused over 40 min) produced a variety of cognitive/affective experiences associated with altered states of consciousness (108). Terms associated with a positive experience were: experiences of unity, spiritual experience, blissful state, insightfulness, complete imagery, audiovisual synesthesia, changed and meaning of precepts; terms associated with a negative experience were: disembodiment, impaired control and cognition, and anxiety; finally, neutral terms (i.e., neither positive nor negative) were: transcendence of time and space, and ineffability (Table 4). Notably, ketamine administration resulted in a decrease in alpha power (as measured by high-density electroencephalography) in the precuneus and the temporoparietal junction (TPJ; based on source analysis) that correlated with measures of dissociation (disembodiment, transcendence of time and space). Changes in activity in the TJP is relevant to the observed affective experience. Anatomically, the human TPJ is commonly described as being situated at the intersection of the posterior end of the superior temporal sulcus, the lateral occipital cortex, and the inferior parietal lobule (IPL) (Figure 3A) (110, 114, 115). The superior aspect of the TPJ overlaps the neighboring IPL, which consists of two major gyri: the supramarginal gyrus (SMG; corresponding to Broadman area (BA) 40) and the angular gyrus (AG; corresponding to BA 39) (115) (Figure 3A). While the TPJ and IPL overlap, they are not synonymous (115). The TPJ participates in a variety of attentional processes (114–117), including bodily self-identification, self-location, first-person perspective (118), and when perturbed, dissociation and the sensation of “out-of-body” experience (119, 120). Intriguingly, in patients with major depressive disorder, functional connectivity between the TPJ and default mode network (DMN) is reduced (121); the importance of this observation is discussed further in Section What Does Functional Imaging Tell Us About Ketamine, Depression, and the Treatment of Chronic Pain?. Highlighting the notion that timing is everything is the observation in mice that ketamine administered 1 week, but not 1 month or 1 h, before administering a contextual fear conditioning (CFC) paradigm exhibited reduced freezing behavior as compared to control animals. In contrast, when administered following CFC or during extinction, ketamine had no effect on subsequent fear expression (122). Thus, the timing of pre-stress event dosing is a critical determinant of its efficacy, and this point must be considered when designing prospective clinical trials in humans.

**Table 4:** Terms and definitions pertaining to altered states of consciousness.

**Term**	**Definition**	**Experience/ mood**
Experiences of unity	Eternal oneness, beyond contradictions, merging of self and environment	Positive
Spiritual experience	Religious cognition, sense of awe, presence of a higher power	Positive
Blissful state	Experiences of boundless pleasure, which may include bliss, peace, and love	Positive
Insightfulness	Profound, clear, original thoughts	Positive
Complex imagery	Vivid complex visual patterns such as scenes and imagery; from past experiences or fantasy, occurring with eyes closed or total darkness	Positive
Elementary imagery	Seeing regular patterns with eyes closed or in total darkness	Positive
Audiovisual synesthesia	Audiovisual abnormalities including shapes, colors of things, or both changing with sounds and noises	Positive
Changed meaning of percepts	Everyday things gain a special and strange meaning; things get more emotionally engaging	Positive
Disembodiment	Floating, being out-of-body, not having a body	Negative
Impaired control and cognition	Cognitive difficulty and disorganization, decreased agency, paralysis, isolation	Negative
Anxiety	Fear, terror, distortion, threat, strangeness	Negative
Transcendence of time and space	Loss of usual sense of time, space, and current location, including being outside of time, no spatial boundaries, and timelessness	-
Ineffability	The experience cannot be adequately described or done justice to with words	-

*Terms and definitions per Vlisides et al. (108)*.

Is the experience of dissociation relevant vis-à-vis ketamine's antidepressant effects? This question was explicitly addressed in a systematic review that examined the relationship between subjective effects induced by a single dose of ketamine and treatment response in patients with major depressive disorder (123). In this analysis, the authors searched the PsycINFO, Embase, and PubMed electronic databases for peer-reviewed journal articles published between 1 January 2000 and 31 May 2019. Per the Methods, “Search terms were combined using logic gates to produce a single query: [((ketamine) OR esketamine)] AND ((((((((((((((((dissociation) OR mystical) OR psychedelic) OR psychotomimetic) OR altered states of consciousness) OR oceanic boundlessness) OR 5D- ASC) OR hallucinogen rating scale) OR phenomenology of consciousness inventory) OR hood's mysticism scale) OR mystical experience questionnaire) OR clinician administered dissociative symptoms scale) OR CADSS) OR BPRS) OR Brief Psychiatric Rating Scale) OR peak experience)) AND ((depression) OR depressive disorder)”. The search retrieved an initial 556 articles; of that number, 343 were duplicates, 203 were determined to be irrelevant or did not otherwise meet inclusion criteria, and 2 were analyses of the same data set, resulting in 8 studies being included in the final analysis. Three of the four largest included studies analyzed (124–126), representing ~55% of the pooled sample, observed a weak to moderate degree of association between the antidepressant and dissociative/hallucinogenic effects of ketamine. While certainly not conclusive, these results preclude summarily dismissing the premise that dissociation is a prerequisite for the efficacy of ketamine as an antidepressant. Intriguingly, recent work has observed an association between ketamine-induced analgesia (in healthy adult volunteers) and dissociation as measured by changes in external perception (as it relates to the misapprehension of external stimuli or surroundings, including body parts, which was obtained from the Bowdle questionnaire, a validated list of 13 items developed to quantify the psychedelic effects of ketamine in healthy volunteers) (127).

Supporting the conjecture that dissociation is relevant to the antidepressant properties of ketamine are the results of Vesuna and colleagues (128). In a technical *tour de force*, the authors made a number of important observations using both rodent models of depression and brain-wide depth electrode recordings from a patient with epilepsy. Administration of a subanesthetic dose of ketamine (50 mg/kg, intraperitoneal) to Thy1-GCaMP6s-expressing mice resulted in the emergence of a slow-wave rhythm (1–3 Hz) in the retrosplenial, but not visual, cortex as measured by wide-field microscopy. These results were recapitulated by phencyclidine (PCP) and dizocilipine (MK-801), both of which are NMDA receptor antagonists that can produce a dissociated state, but not by memantine (also an NMDA receptor antagonist that produces dissociation) or lysergic acid diethylamide (LSD). These results point to mechanisms beyond NMDA receptors in driving this retrosplenial slow-wave rhythm. Using two photon Ca^2+^ imaging and engineered mouse lines in which GCaMP6m was restricted to specific cortical layers (Cux2-CreER for layer 2/3 and Rbp4-Cre for layer 5), they next demonstrated that the slow retrosplenial rhythm arose in layer 5. In parallel, 32-channel single unit electrophysiology recordings from deep brain regions revealed correlation of spike firing between the retrosplenial cortex and nearly all brain regions prior to ketamine administration. Following ketamine administration there was a marked reduction in spike correlation between the retrosplenial cortex and somatosensory cortex, subiculum, ventral/anteromedial thalamus, and the red nucleus; in contrast, the correlation in spike firing increased between the laterodorsal thalamus and the anteroventral thalamus and those regions with the retrosplenial cortex. At the behavioral level, ketamine had no effect on reflexive behavior (as measured by paw-flick on a hot-plate test) but it abolished affective/emotional (as measured by paw-lick) and motivational (as measured by jump-to-escape test) behaviors. With regards to escape and social interactions, ketamine suppressed both tail-suspension escape responses and resident-intruder interactions. Ketamine-induced changes in affective behaviors were dose dependent such that they were preserved at a dose of 13 mg/kg but not at 25 mg/kg. Correspondingly, the ketamine-induced slow wave oscillation (1–3 Hz) observed using wide-field and two photon imaging was again detected in the retrosplenial cortex using *in situ* fiber photometry at the 25, but not 13, mg/kg dose, strongly suggesting that this slow rhythm was causally linked to ketamine-induced dissociation. Importantly, the pattern of behavioral responses induced by ketamine were not recapitulated by other classes of drugs (analgesics: buprenorphine, lidocaine; hallucinogens: LSD; anxiolytics: diazepam) known to effect subsets of these effects; the only drug that mirrored ketamine was PCP.

But is the ketamine-induced slow wave rhythm necessary and sufficient to induce dissociative behaviors? To answer this question, layer 5 neurons in the retrosplenial cortex were optogenetically activated at 2 Hz in eNpHR3.0/CHR2 (eNPAC) mice (128). Dissociation-like (i.e., affective paw-licking, time to escape, rearing, tail-suspension escape behavior), but not reflexive (nor changes in righting-reflex or open-field), behaviors were observed in response to low frequency stimulation; stimulating other cortical regions in which eNPAC was also expressed (deep somatosensory cortex) failed to induce dissociative behaviors. Thus, a slow-wave rhythm in layer 5 retrosplenial cortical neurons, whether induced by ketamine or optogenetic activation, appears to be causal with respect to the disconnect between sensory and affective behaviors.

We now return to the role of HCN channels in mediating the antidepressant effects of ketamine. If the retrosplenial slow wave rhythm is necessary to induce dissociation, what is the molecular basis of that rhythm? As suggested by the results of Zhang et al. (93) discussed above, one plausible target are HCN channels. As noted previously, HCN channels and are present throughout the nervous system, including the retrosplenial cortex (31, 32). The molecular underpinning of the slow rhythm was explicitly examined using gene targeting strategies in which HCN1 or NMDA receptor expression was selectively disrupted in retrosplenial cortical neurons (*via* local injection of AAVdj-Ef1a-Cre and AAVdj-Ef1a-DIO-GCaMP6m viruses into the retrosplenial cortex of adult homozygous floxed-channel [HCN1 or GRIN1] transgenic mice) (128). In both groups of animals, ketamine-induced slow-wave oscillations were significantly reduced as compared to wild-type mice. Correspondingly, ketamine again abolished affective (paw-licking) but not reflexive (paw-flicking) behaviors in floxed mice whereas the affective response was restored in response to injection of the Cre virus into the retrosplenial cortex of HCN1-floxed mice. In aggregate, these results firmly establish a role for ketamine mediated-inhibition of HCN1 channels in both the retrosplenial slow-wave rhythm and the accompanying behavioral dissociation.

In humans, the retrosplenial cortex is one region within the posterior cingulate cortex, which in turn is part of the posteromedial cortex (PMC), and the PMC is thought to be a homologous structure to the rodent retrosplenial cortex (129). The PMC consists of: Brodmann area (BA) 7m (mesial parietal area of the precuneus), BA 23a, b, c (posterior cingulate), BA 29–30 (retrosplenial cortex), and BA 31 (transition zone between BA 7m and 23c (Figure 3B) (111, 130–132). As a core node in the default mode network (DMN), changes in activity in the PMC have been linked to various neuropsychiatric condition (e.g., Alzheimer's disease/mild cognitive impairment, schizophrenia, depression and anxiety, epilepsy, autism spectrum disorder, attention deficit/hyperactivity disorder) as well as to self-referential thinking (111, 132, 133).

The relationship between the retrosplenial slow-wave rhythm and the behavioral dissociation observed in mice was also examined in a human patient with epilepsy using brain-wide intracranial electrodes that enabled intracranial electroencephalography/stereoelectroencephalography for both diagnostic recording and focal stimulation (128, 134). During pre-seizure auras, a distinct slow-wave (3.4 Hz) patten of activity was observed in the PMC bilaterally. During these auras, the patient reported an experience highly reminiscent of a dissociated state (135) as suggested by the following comments:

“*I was listening to two parts of my brain speak to each other in a way that a third part of my brain, which I considered to be me, was able to listen.”*

“*What would it feel like if someone else were to come into your head?… What I considered me shrank to this other part of me where the other parts of my brain that were talking, I stopped considering them me.”*

“*…where in this 3D space am I?… I took a blanket…I threw it over my body, just to see, because I knew that when I don't feel it, I don't consider it me and immediately my legs were no longer a part of me..*.

Critically, brief stimulation (50 Hz, 2–10 mA, total duration 1.3 ± 0.47 s; mean ± SEM) of foci associated with seizure activity in the right PMC evoked a report of a dissociative aura-like experience resembling that which preceded seizure onset. Comments reported during stimulation of the right PMC included:

“*Felt similar to the seizure beginning.”*

“*Felt like an aura.”*

“*It's like I'm about to have a seizure.”*

Similarly, the same stimulation protocol applied to the left PMC also produced a sense of dissociation, albeit one without the negative overtones of an impending seizure. Comments reported during stimulation of the left PMC included:

“*This feeling of being disconnected from something…that was a little pleasant”*.

“*It's like being weightless in your own mind as a personality.”*

“…*maybe the same way a pilot can lose control of a plane. Like they can be forced out of the cockpit or to not control it… but still see what's happening to the whole plane, that's kinda what just happened. I got pulled out of…the pilot's chair, but I could still see all the gauges… You can see the information flowing – you can't control it, but you can see it.”*

Together, the mouse and human data provide a compelling argument for ketamine-mediated inhibition of HCN1 channels as the basis for deep brain slow-wave oscillatory activity as the genesis of the dissociated behavioral state. It is important to note here that the results reported by Vlisides et al. describing a role for the TPJ and precuneus in underlying the dissociative effects of ketamine (108) are not discordant with the role proposed for the PMC by Vesuna et al. (128) as both TPJ and PMC are adjacent brain regions (Figure 3C) and are critical nodes in the default mode network (DMN) (115, 132), and as such, are functionally linked (Figure 3D).

Highlighting a role for ketamine-mediated inhibition of HCN channels as underling ketamine's antidepressant/dissociative effects is provided in the study by Anand et al. (136). Here, (R,S)-ketamine (0.26 mg/kg, iv bolus followed by a 90-min infusion of 0.65 mg/kg) was administered to healthy volunteers (*n* = 16) after having been administered lamotrigine (300 mg, oral dose) or placebo 2 h prior to receiving ketamine. Lamotrigine (Lamictal^®^ GlaxoSmithKline, Brentford, UK) is an antiepileptic drug with a complex mechanism of action that includes inhibition of Na_V_, Ca_V_2.1 and Ca_V_2.2 channels (137); interestingly, however, lamotrigine increases HCN-dependent current I_h_ amplitude at or near resting membrane potential and markedly shifts the V_1/2_ to a more depolarized value (~-83 and ~-11 mV, control and lamotrigine, respectively) (138). After 60 min of ketamine administration, plasma levels were ~155 ng/ml [~0.65 μM, which is comparable to that required for its antidepressant effects in patients with treatment-resistant major depression (19)]. As expected, ketamine increased dissociative symptoms (as measured by the Clinician-Administered Dissociative States Scale, CADSS, which assesses symptoms of dissociation—notably, impairments in body, environmental, and time perception, memory impairment, and feelings of unreality) and pretreatment with lamotrigine significantly attenuated this effect. Similarly, ketamine significantly increased the positive symptoms of schizophrenia score (as measured by the Brief Psychiatric Rating Scale (BPRS) positive symptoms subscale), and this effect, too, was limited by lamotrigine. Other ketamine-induced changes in neuropsychiatric/neurobehavioral measures were also significantly blunted. These results, along with those of Vesuna et al. (128), underscore not only the well-known dissociative properties of subanesthetic ketamine, but that such dissociation is HCN-channel dependent.

## What Does Functional Imaging Tell Us About Ketamine, Depression, and the Treatment of Chronic Pain?

### The Default Mode Network (DMN)–Anatomic and Functional Considerations

The human brain decodes, interprets, and creates meaning of the external world by means of functional networks that connect disparate brain regions (139–146). It has long been known that functional networks map onto known large-scale brain systems, including the motor (147), auditory (148), and visual systems (149), as well as higher-level systems, such as those for executive control (150). Numerous networks have been identified and include: dorsal attention (DAN), fronto-parietal (FPN), parietal memory (PMN), salience (Sal), default mode (DMN), contextual association (CAN), ventral attention (VAN), medial visual (mVis), lateral visual (lVis), leg somatomotor (lSM), face somatomotor (fSM), hand somatomotor (hSM), auditory (Aud), premotor (PMot), and cingulo-opercular (CON) (151). The DMN, which is comprised of the medial prefrontal cortex (MPFC), medial temporal lobes (MTLs), and posterior cingulate cortex (PCC)/retropslenial cortex (RSC) (145, 152), is preferentially activated when an individual is not engaged with the external world (153); it is what the brain does when it is not specifically focused on doing anything else but is considering oneself [ex., remembering, considering hypothetical social interactions, thinking of one's own future (153)]. Perturbations in these networks, including the DMN, are often associated with disorders of thought and consciousness (133, 153–158). What, then, does ketamine do to the DMN?

### Ketamine Disrupts DMN Connectivity to Other Brain Networks

In healthy adult volunteers, (S)-ketamine (0.25 mg/kg infused over 45 min) decreases functional connectivity of the DMN to the “dorsal nexus” [DN; identified as a “bilateral dorsal medial prefrontal cortex region showing dramatically increased depression-associated functional connectivity with large portions of a cognitive control network (CCN), the default mode network (DMN), and a rostral affective network (AN)” and to the pregenual anterior cingulate (PACC) and medioprefrontal cortex (MPFC) *via* the posterior cingulate cortex (PCC)] (159). Notably, the breakdown of the DMN that is observed at “light to moderate” levels of (R,S)-ketamine-induced sedation [median (range)–Ramsay Scale (RS): 2 [2–3], University of Michigan Sedation Scale (USMMS): 1.5 [0–2]: RS: 1 = anxious, agitated, or both; 2 = cooperative, oriented, and calm; 3 = response to command only, clear but slow; 4 = evident response to glabellum stimulation or to loud auditory stimulation; 5 = slow response to glabellum stimulation or to loud auditory stimulation; and 6 = no response; UMSS: 0 = awake and alert; 1 = lightly sedated, tired, appropriate response to conversation and/or to sounds; 2 = moderate sedation, drowsy, easily aroused by tactile stimulation; 3 = deep sedation, deep sleep, aroused only by strong physical stimulation; and 4 = impossible to arouse) are rather unique, with tenuous, if any, effects on other networks, including executive control, salience, auditory, and visual (160).

Correspondingly, (S)-ketamine, at estimated plasma concentrations between 0.25 and 0.48 μg/ml (1.1–2.0 μM), increased connectivity between the executive control network (ECN) and parts of the anterior cingulum and superior frontal gyrus but decreased connectivity between the salience network (SN) and the calcarine fissure. Subjects (*n* = 17, healthy adult males) routinely reported feelings of “oceanic boundlessness” (i.e., dissolution of ego boundaries associated with positive emotions), “dread of ego dissolution” (a distressing experience of depersonalization, thought disorder and loss of body control), “visionary restructuralization” (e.g., visual illusions and hallucinations), “auditory alterations” (e.g., illusions and hallucinations) and “vigilance reduction.” Interestingly, only visionary restructuralization correlated with changes in a single network (DMN) (161). When thinking about the relevance of these observations vis-à-vis the relationship between subjective effects induced by a single dose of ketamine and treatment response (108, 123, 128), it is important to remember that the ketamine concentrations here are comparable to those achieved in subjects with major depressive illness (Table 2). The subjective experiences reported here are not dissimilar to the dissociated state reported during pre-seizure auras and in response to electrical stimulation of the PMC in the patient with epilepsy (128, 134), and emphasize the commonality and potential relevance of the experience to symptomatic relief in patients with depression.

### Ketamine Disrupts Depression-Associated Patterns of Functional Connectivity

But what impact does ketamine have on network function in individuals with depression? In patients with post-traumatic stress disorder (PTSD; *n* = 11, 10 female), (R,S)-ketamine (0.5 mg/kg) significantly improved symptoms of PTSD and depression; the improvement was greater than that achieved with the anxiolytic midazolam, indicating that simply relieving the anxiety component of their symptoms was insufficient to achieve maximal benefit (162). Symptomatic improvement correlated with an increase in functional connectivity between the ventromedial (vm)PFC (and as noted the PFC is part of the DMN) and the amygdala [a brain region fundamental to anxiety (163)]. These results agree with other studies in which it was demonstrated that ketamine-induced improvements in symptom severity in subjects with treatment-resistant depression were associated with network-dependent changes in connectivity (both increased and decreased) during emotional processing (164–167). Previous work has shown that major depressive disorder is associated with hyperconnectivity within the DMN (168, 169), and it has been postulated that the antidepressant efficacy of ketamine hinges on its ability to “[reverse] the maladaptive affective- and default mode network–driven hyperconnectivity that typifies depression” (170). But simply examining changes in functional magnetic resonance imaging (fMRI) activity as measured by blood-oxygen-level dependent (BOLD) signals in single (or even multiple) networks may not tell the whole story (139, 171).

Neurons generate action potentials (both spontaneous and stimulus-evoked) in response to membrane depolarization, which in turn give rise to local field potentials (LFPs). LFPs can be measured at the scalp using electroencephalography, and as they are continuously present can be organized by frequency (in Hz): slow, < 1; δ, 1–4; θ, 5–8, α, 9–12; α, 13–25; γ, 26–80 (172). It is thought that the complex interplay of these rhythms is the basis for attention and cognition (173, 174), that when disrupted, results in disorders in thought and attention (175). These rhythms can be observed using multiple approaches, including magnetoencephalography (MEG) (176). In subjects with major depressive disorder (MDD), (R,S)-ketamine (0.5 mg/kg infused over 40 min) was again observed to improve symptoms; connectivity was measured using MEG in both healthy volunteers and individuals with MDD. Applying connectivity analysis (wherein the correlation between waves is examined), MDD subjects who responded to ketamine displayed increased cross-frequency connectivity in δ-α and δ-γ bands whereas MDD non-responders (and healthy controls) showed a decrease in connectivity (177). These results suggest not only that there may be functional subtypes of MDD but reinforce the proposition that ketamine-modulation of δ oscillations is instrumental in mediating its beneficial effect.

### The Interface Between Depression, Brain Dynamics, and Ketamine-Induced Neuropathic Pain Relief

This then brings us to the final question as to the impact of ketamine on functional connectivity and the consequences of any such modulation on neuropathic pain. In adults with diverse etiology (traumatic injury, postherpetic neuralgia, vasculitis, stroke, spinal cord injury, scleroderma, syringomelia) neuropathic pain, (R,S)-ketamine (0.5–2.0 mg/kg/hr for 6 h/day over 5 consecutive days (mean dose 1.1 mg/kg/hr) with dose individually titrated to achieve maximal pain relief (while minimizing adverse effects to a tolerable level) significantly improved pain scores (measured using thermal quantitative testing) in 14/30 (46.7%) of subjects (“Responders”) while 16/30 (53.3%) did not experience improvement (“Non-Responders”) (178). In contrast to healthy control subjects, both Responders and Non-Responders had significantly higher anxiety, depression, and pain catastrophizing scores but significantly lower resiliency scores. Along similar lines, pain rumination (a subscale in the Pain Catastrophizing Scale) in patients with chronic pain (but not in healthy controls) was positively correlated to mPFC functional connectivity with posterior cingulate cortex/precuneus, retropslenial cortex, medial thalamus, and periaqueductal gray/periventricular gray (179). These data are concordant with the observation that chronic postsurgical pain is significantly associated with depression, anxiety, catastrophizing, mental health, kinesiophobia, and self-efficacy (89). At baseline (i.e., pre-ketamine administration), Responders had greater connectivity between the DMN and the descending antinociceptive pathway [which is comprised of the prefrontal cortex, subgenual anterior cingulate cortex (sgACC), periaqueductal gray (PAG), and the rostral ventromedial medulla (RVM) (180, 181)] than did Non-Responders or healthy controls, and the magnitude of connectivity correlated with percentage of pain relief (178). In contrast to the greater strength of the connection between the DMN and descending pathway in subjects with neuropathic pain who experience pain relief in response to ketamine, other subjects with neuropathic pain and who respond to ketamine have significantly lower functional connectivity between the mPFC and precuneus (182). Opposing directional changes in connectivity only serve to reinforce the premise that the “pain connectome” is a dynamic construct (183), one which can be modified (184).

Finally, Kucyi et al. (181) examined the relationship between antinociceptive and DMN networks during “mind wandering.” What they found was the following:

“*(i) pain-induced default mode network (DMN) deactivations were attenuated during mind wandering away from pain;*

*(ii) functional connectivity fluctuations between the DMN and periaqueductal gray (PAG) dynamically tracked spontaneous attention away from pain; and*

*(iii) across individuals, stronger PAG–DMN structural connectivity and more dynamic resting state PAG–DMN functional connectivity were associated with the tendency to mind wander away from pain.”*

Notably, “mind wandering” has been defined as a state of “perceptual decoupling,” or “disengagement of attention from perception” (181). Conceptually, this may not be all that different from many of the terms associated with ketamine-induced altered states of consciousness (Table 4) or of an out-of-body experience.

## Conclusion

Previously conducted meta-analyses indicated that evidence of efficacy for ketamine in preventing the development of CPSP was lacking (185, 186). However, more recent meta-analyses and guidelines indicate that ketamine can reduce or prevent the development of CPSP (1, 5). These disparate results may be explained, at least in part, by the multiple molecular targets through which ketamine can exert its effects on both acute and chronic pain. While NMDA may be presumed to be the primary mediator, non-NMDA targets, such as HCN1 channels, may in fact be critical. The retrosplenial slow-wave rhythm elicited by subanesthetic levels of ketamine is not driven by its NMDA activity, and the relationship between this slow-wave rhythm and the behavioral dissociation is clear.

Much remains to be determined about the precise means by which subanesthetic ketamine alters functional brain connectivity and how this impacts chronic and neuropathic pain; however, the emerging data from both preclinical animal studies and human clinical studies clearly implicates changes in brain activity that result in a sense of dissociation. Given these results, subanesthetic doses of ketamine *prior* to anesthetic induction (or following full recovery of consciousness postoperatively) may be necessary to elicit this effect. If the hypothesis that induction and awareness of the dissociated state is a necessary feature for the antidepressant effects of ketamine, and that it is those effects which are critical in preventing the development of chronic postsurgical pain, then several points should be remembered: 1) roughly 50% of patients with major depressive disorder/treatment resistant depression experience symptomatic relief from ketamine, which will necessitate increasing sample size, and 2) fMRI may more accurately identify patients at risk given baseline differences in connectivity between ketamine responders and non-responders, and the use of fMRI as a screening tool may result in selection of an appropriate patient population.

It is quite possible that ketamine will not prevent the development of CPSP in all patients. Identifying those patients in whom ketamine may prove beneficial will be necessary in future studies. Those studies should at least assess known preoperative risk factors (i.e., depression, anxiety, catastrophizing, kinesiophobia, self-efficacy) and stratify accordingly. While numerous reports have suggested that fMRI studies might also serve as a suitable biomarker for identifying those individuals at risk for CPSP, as well as those who might derive benefit from ketamine, the routine incorporation of fMRI studies for this purpose is not realistic for logistical (i.e., need to obtain the study and have results interpreted preoperatively) and economic (they are expensive) reasons. Results from EEG studies suggest that increases in connectivity, most notably in δ-α and δ-γ bands, might be a suitable predictive biomarker; whether such changes can be easily detected using simple bifrontal scalp EEG measurements (as can be obtained using commercially available processed EEG devices such as the Masimo SedLine^®^ or the Medtronic BIS™ brain monitoring system) would need to be determined. Rigorously designed prospective clinical trials are needed to test these hypotheses before attempting to incorporate these approaches into routine clinical practice.

## Author Contributions

DW and PG contributed equally to the concept and writing/editing of the manuscript. All authors contributed to the article and approved the submitted version.

## Funding

This work was supported by the Department of Anesthesiology, Weill Cornell Medicine (PG) and the Burke Neurological Institute (DW).

## Conflict of Interest

PG is a co-inventor on patents related to the development of alkylphenols for the treatment of neuropathic pain and both PG and DW serve on the Scientific Advisory Board for Akelos, Inc., a research-based biotechnology company that has secured a licensing agreement for the use of those patents.

## Publisher's Note

All claims expressed in this article are solely those of the authors and do not necessarily represent those of their affiliated organizations, or those of the publisher, the editors and the reviewers. Any product that may be evaluated in this article, or claim that may be made by its manufacturer, is not guaranteed or endorsed by the publisher.

## References

[1] Schwenk ES Viscusi ER Buvanendran A Hurley RW Wasan AD Narouze S. Consensus guidelines on the use of intravenous ketamine infusions for acute pain management from the American society of regional anesthesia and pain medicine, the American academy of pain medicine, and the American society of anesthesiologists. Reg Anesth Pain Med. (2018) 43:456–66. 10.1097/AAP.0000000000000806 29870457 PMC6023582

[2] Brinck EC Tiippana E Heesen M Bell RF Straube S Moore RA. Perioperative intravenous ketamine for acute postoperative pain in adults. Cochrane Database Syst Rev. (2018) 12:CD012033. 10.1002/14651858.CD012033.pub4 30570761 PMC6360925

[3] Brinck ECV Maisniemi K Kankare J Tielinen L Tarkkila P Kontinen VK. Analgesic effect of intraoperative intravenous S-ketamine in opioid-naive patients after major lumbar fusion surgery is temporary and not dose-dependent: a randomized, double-blind, placebo-controlled clinical trial. Anesth Analg. (2021) 132:69–79. 10.1213/ANE.0000000000004729 32167978

[4] Cohen SP Bhatia A Buvanendran A Schwenk ES Wasan AD Hurley RW. Consensus guidelines on the use of intravenous ketamine infusions for chronic pain from the American society of regional anesthesia and pain medicine, the American academy of pain medicine, and the american society of anesthesiologists. Reg Anesth Pain Med. (2018) 43:521–46. 10.1097/AAP.0000000000000808 29870458 PMC6023575

[5] Orhurhu V Orhurhu MS Bhatia A Cohen SP. Ketamine infusions for chronic pain: a systematic review and meta-analysis of randomized controlled trials. Anesth Analg. (2019) 129:241–54. 10.1213/ANE.0000000000004185 31082965

[6] Michelet D Brasher C Horlin AL Bellon M Julien-Marsollier F Vacher T. Ketamine for chronic non-cancer pain: a meta-analysis and trial sequential analysis of randomized controlled trials. Eur J Pain. (2018) 22:632–46. 10.1002/ejp.1153 29178663

[7] Fda. Approves New Nasal Spray Medication for Treatment-Resistant Depression. Available Only at a Certified Doctor's Office or Clinic: Food and Drug Administration (2019). Available online at: https://www.fda.gov/news-events/press-announcements/fda-approves-new-nasal-spray-medication-treatment-resistant-depression-available-only-certified.

[8] Spravato (Esketamine): European Medicines Agency. Available online at: https://www.ema.europa.eu/en/medicines/human/EPAR/spravato.

[9] Zanos P Moaddel R Morris PJ Riggs LM Highland JN Georgiou P. Ketamine and ketamine metabolite pharmacology: insights into therapeutic mechanisms. Pharmacol Rev. (2018) 70:621–60. 10.1124/pr.117.015198 29945898 PMC6020109

[10] Rao LK Flaker AM Friedel CC Kharasch ED. Role of cytochrome P4502b6 polymorphisms in ketamine metabolism and clearance. Anesthesiology. (2016) 125:1103–12. 10.1097/ALN.0000000000001392 27763887

[11] Lumsden EW Troppoli TA Myers SJ Zanos P Aracava Y Kehr J. Antidepressant-Relevant concentrations of the ketamine metabolite (2R6R)-Hydroxynorketamine do not block NMDA receptor function. Proc Natl Acad Sci USA. (2019) 116:5160–9. 10.1073/pnas.1816071116 30796190 PMC6421428

[12] Little B Chang T Chucot L Dill WA Enrile LL Glazko AJ. Study of ketamine as an obstetric anesthetic agent. AmJ Obstetrics Gynecol. (1972) 113:247–60. 10.1016/0002-9378(72)90774-0 4554554

[13] Grant IS Nimmo WS McNicol LR Clements JA. Ketamine disposition in children and adults. Br J Anaesth. (1983) 55:1107–11. 10.1093/bja/55.11.1107 6639827

[14] Sleigh J Pullon RM Vlisides PE Warnaby CE. Electroencephalographic slow wave dynamics and loss of behavioural responsiveness induced by ketamine in human volunteers. Br J Anaesth. (2019) 123:592–600. 10.1016/j.bja.2019.07.021 31492526 PMC6871266

[15] White PF Schuttler J Shafer A Stanski DR Horai Y Trevor AJ. Comparative pharmacology of the ketamine isomers. Studies in volunteers. Br J Anaesth. (1985) 57:197–203. 10.1093/bja/57.2.197 3970799

[16] Clements JA Nimmo WS. Pharmacokinetics and analgesic effect of ketamine in man. Br J Anaesth. (1981) 53:27–30. 10.1093/bja/53.1.27 7459184

[17] Grant IS Nimmo WS Clements JA. Pharmacokinetics and analgesic effects of I.M. And oral ketamine. Br J Anaesth. (1981) 53:805–10. 10.1093/bja/53.8.805 7272143

[18] Clements JA Nimmo WS Grant IS. Bioavailability, pharmacokinetics, and analgesic activity of ketamine in humans. J Pharmaceut Sci. (1982) 71:539–42. 10.1002/jps.2600710516 7097501

[19] Farmer CA Gilbert JR Moaddel R George J Adeojo L Lovett J. Ketamine metabolites, clinical response, and gamma power in a randomized, placebo-controlled, crossover trial for treatment-resistant major depression. Neuropsychopharmacology. (2020) 45:1398–404. 10.1038/s41386-020-0663-6 32252062 PMC7297997

[20] Perez-Ruixo C Rossenu S Zannikos P Nandy P Singh J Drevets WC. Population pharmacokinetics of esketamine nasal spray and its metabolite Noresketamine in healthy subjects and patients with treatment-resistant depression. Clin Pharmacokinet. (2021) 60:501–16. 10.1007/s40262-020-00953-4 33128208

[21] Zarate CA Jr Brutsche N Laje G Luckenbaugh DA Venkata SL. Relationship of ketamine's plasma metabolites with response, diagnosis, and side effects in major depression. Biol Psychiatry. (2012) 72:331–8. 10.1016/j.biopsych.2012.03.004 22516044 PMC3442255

[22] Goldstein PA. HCN1 channels as targets for volatile anesthetics: coming to the fore. Anesth Analg. (2015) 121:594–6. 10.1213/ANE.0000000000000871 26287292

[23] Chen X Shu S Bayliss DA. HCN1 channel subunits are a molecular substrate for hypnotic actions of ketamine. J Neurosci. (2009) 29:600–9. 10.1523/JNEUROSCI.3481-08.2009 19158287 PMC2744993

[24] Zhou C Douglas JE Kumar NN Shu S Bayliss DA Chen X. Forebrain hcn1 channels contribute to hypnotic actions of ketamine. Anesthesiology. (2013) 118:785–95. 10.1097/ALN.0b013e318287b7c8 23377220 PMC3605219

[25] Biel M Wahl-Schott C Michalakis S Zong X. Hyperpolarization-Activated cation channels: from genes to function. Physiol Rev. (2009) 89:847–85. 10.1152/physrev.00029.2008 19584315

[26] Wahl-Schott C Biel M. HCN channels: structure, cellular regulation and physiological function. Cell Mol Life Sci. (2009) 66:470–94. 10.1007/s00018-008-8525-0 18953682 PMC11131499

[27] Robinson RB Siegelbaum SA. Hyperpolarization-Activated cation currents: from molecules to physiological function. Annu Rev Physiol. (2003) 65:453–80. 10.1146/annurev.physiol.65.092101.142734 12471170

[28] He C Chen F Li B Hu Z. Neurophysiology of HCN channels: from cellular functions to multiple regulations. Prog Neurobiol. (2014) 112:1–23. 10.1016/j.pneurobio.2013.10.001 24184323

[29] Santoro B Shah MM. Hyperpolarization-activated cyclic nucleotide-gated channels as drug targets for neurological disorders. Ann Rev Pharmacol Toxicol. (2020) 60:109–31. 10.1146/annurev-pharmtox-010919-023356 31914897

[30] Much B Wahl-Schott C Zong X Schneider A Baumann L Moosmang S. Role of subunit heteromerization and *N*-linked glycosylation in the formation of functional hyperpolarization-activated cyclic nucleotide-gated channels. J Biol Chem. (2003) 278:43781–6. 10.1074/jbc.M306958200 12928435

[31] Moosmang S Biel M Hofmann F Ludwig A. Differential distribution of four hyperpolarization-activated cation channels in mouse brain. Biol Chem. (1999) 380:975–80. 10.1515/BC.1999.121 10494850

[32] Notomi T Shigemoto R. Immunohistochemical localization of i_h_ channel subunits, Hcn1-4, in the rat brain. J Comp Neurol. (2004) 471:241–76. 10.1002/cne.11039 14991560

[33] Chen S Wang J Siegelbaum SA. Properties of hyperpolarization-activated pacemaker current defined by coassembly of HCN1 and HCN2 subunits and basal modulation by cyclic nucleotide. J Gen Physiol. (2001) 117:491–504. 10.1085/jgp.117.5.491 11331358 PMC2233656

[34] Gao LL McMullan S Djouhri L Acosta C Harper AA Lawson SN. Expression and properties of hyperpolarization-activated current in rat dorsal root ganglion neurons with known sensory function. J Physiol. (2012) 590(Pt 19):4691–705. 10.1113/jphysiol.2012.238485 22753545 PMC3487031

[35] Ku SM Han MH. Hcn channel targets for novel antidepressant treatment. Neurotherapeutics. (2017) 14:698–715. 10.1007/s13311-017-0538-7 28560710 PMC5509632

[36] Kim CS Johnston D. A possible link between Hcn channels and depression. Chronic Stress (Thousand Oaks). (2018) 2:2470547018787781. 10.1177/2470547018787781 30259006 PMC6152912

[37] Hirota K Hashimoto Y Lambert DG. Interaction of intravenous anesthetics with recombinant human M1-M3 muscarinic receptors expressed in chinese hamster ovary cells. Anesth Analg. (2002) 95:1607–10. 10.1097/00000539-200212000-00025 12456425

[38] Yamakura T Chavez-Noriega LE Harris RA. Subunit-dependent inhibition of human neuronal nicotinic acetylcholine receptors and other ligand-gated ion channels by dissociative anesthetics ketamine and dizocilpine. Anesthesiology. (2000) 92:1144–53. 10.1097/00000542-200004000-00033 10754635

[39] Moaddel R Abdrakhmanova G Kozak J Jozwiak K Toll L Jimenez L. Sub-anesthetic concentrations of (R,S)-ketamine metabolites inhibit acetylcholine-evoked currents in α7 nicotinic acetylcholine receptors. Eur J Pharmacol. (2013) 698:228–34. 10.1016/j.ejphar.2012.11.023 23183107 PMC3534778

[40] Kapur S Seeman P. Nmda receptor antagonists ketamine and pcp have direct effects on the dopamine D(2) and serotonin 5-HT(2) receptors-implications for models of schizophrenia. Mol Psychiatry. (2002) 7:837–44. 10.1038/sj.mp.4001093 12232776

[41] Roth BL Gibbons S Arunotayanun W Huang XP Setola V Treble R. The ketamine analogue methoxetamine and 3- and 4-methoxy analogues of phencyclidine are high affinity and selective ligands for the glutamate nmda receptor. PLoS ONE. (2013) 8:e59334. 10.1371/journal.pone.0059334 23527166 PMC3602154

[42] Can A Zanos P Moaddel R Kang HJ Dossou KS Wainer IW. Effects of ketamine and ketamine metabolites on evoked striatal dopamine release, dopamine receptors, and monoamine transporters. J Pharmacol Exp Ther. (2016) 359:159–70. 10.1124/jpet.116.235838 27469513 PMC5034706

[43] Jordan S Chen R Fernalld R Johnson J Regardie K Kambayashi J. *In vitro* biochemical evidence that the psychotomimetics phencyclidine, ketamine and dizocilpine (MK-801) are inactive at cloned human and rat dopamine D2 receptors. Eur J Pharmacol. (2006) 540:53–6. 10.1016/j.ejphar.2006.04.026 16730695

[44] Ho MF Correia C Ingle JN Kaddurah-Daouk R Wang L Kaufmann SH. Ketamine and ketamine metabolites as novel estrogen receptor ligands: induction of cytochrome P450 and AMPA glutamate receptor gene expression. Biochem Pharmacol. (2018) 152:279–92. 10.1016/j.bcp.2018.03.032 29621538 PMC5960634

[45] Morris PJ Moaddel R Zanos P Moore CE Gould TD Zarate CA. Synthesis and N-methyl-D-aspartate (NMDA) receptor activity of ketamine metabolites. Org Lett. (2017) 19:4572–5. 10.1021/acs.orglett.7b02177 28829612 PMC5641405

[46] Parsons CG Quack G Bresink I Baran L Przegalinski E Kostowski W. Comparison of the potency, kinetics and voltage-dependency of a series of uncompetitive NMDA receptor antagonists *in vitro* with anticonvulsive and motor impairment activity *in vivo*. Neuropharmacology. (1995) 34:1239–58. 10.1016/0028-3908(95)00092-K 8570022

[47] Hirota K Okawa H Appadu BL Grandy DK Devi LA Lambert DG. Stereoselective interaction of ketamine with recombinant mu, kappa, and delta opioid receptors expressed in Chinese hamster ovary cells. Anesthesiology. (1999) 90:174–82. 10.1097/00000542-199901000-00023 9915326

[48] Hirota K Sikand KS Lambert DG. Interaction of ketamine with mu_2_ opioid receptors in sh-sy5y human neuroblastoma cells. J Anesth. (1999) 13:107–9. 10.1007/s005400050035 14530949

[49] Nemeth CL Paine TA Rittiner JE Beguin C Carroll FI Roth BL. Role of kappa-opioid receptors in the effects of salvinorin a and ketamine on attention in rats. Psychopharmacology (Berl). (2010) 210:263–74. 10.1007/s00213-010-1834-7 20358363 PMC2869248

[50] Robson MJ Elliott M Seminerio MJ Matsumoto RR. Evaluation of Sigma (Σ) receptors in the antidepressant-like effects of ketamine *in vitro* and *in vivo*. Eur Neuropsychopharmacol. (2012) 22:308–17. 10.1016/j.euroneuro.2011.08.002 21911285

[51] Nishimura M Sato K Okada T Yoshiya I Schloss P Shimada S. Ketamine inhibits monoamine transporters expressed in human embryonic kidney 293 cells. Anesthesiology. (1998) 88:768–74. 10.1097/00000542-199803000-00029 9523822

[52] Zhao Y Sun L. Antidepressants modulate the *in vitro* inhibitory effects of propofol and ketamine on norepinephrine and serotonin transporter function. J Clin Neurosci. (2008) 15:1264–9. 10.1016/j.jocn.2007.11.007 18815045 PMC2605271

[53] Todorovic SM Perez-Reyes E Lingle CJ. Anticonvulsants but not general anesthetics have differential blocking effects on different t-type current variants. Mol Pharmacol. (2000) 58:98–108. 10.1124/mol.58.1.98 10860931

[54] Denson DD Eaton DC. Ketamine inhibition of large conductance Ca^2+^-activated K^+^ channels is modulated by intracellular Ca^2+^. Am J Physiol. (1994) 267(5 Pt 1):C1452–8. 10.1152/ajpcell.1994.267.5.C1452 7977705

[55] Ko SH Lee SK Han YJ Choe H Kwak YG Chae SW. Blockade of myocardial ATP-sensitive potassium channels by ketamine. Anesthesiology. (1997) 87:68–74. 10.1097/00000542-199707000-00010 9232136

[56] Dreixler JC Jenkins A Cao YJ Roizen JD Houamed KM. Patch-clamp analysis of anesthetic interactions with recombinant SK2 Subtype neuronal calcium-activated potassium channels. Anesth Analg. (2000) 90:727–32. 10.1097/00000539-200003000-00040 10702465

[57] Haeseler G Tetzlaff D Bufler J Dengler R Munte S Hecker H. Blockade of voltage-operated neuronal and skeletal muscle sodium channels by S(+)- and R(-)-ketamine. Anesth Analg. (2003) 96:1019–26. 10.1213/01.ANE.0000052513.91900.D5 12651652

[58] Sleigh J Harvey M Denny B. Ketamine-more mechanisms of action than just nmda blockade. Trends Anaesthesia Crit Care. (2014) 4:76–81. 10.1016/j.tacc.2014.03.002

[59] Jouguelet-Lacoste J La Colla L Schilling D Chelly JE. The use of intravenous infusion or single dose of low-dose ketamine for postoperative analgesia: a review of the current literature. Pain Med. (2015) 16:383–403. 10.1111/pme.12619 25530168

[60] Wang L Johnston B Kaushal A Cheng D Zhu F Martin J. Ketamine added to morphine or hydromorphone patient-controlled analgesia for acute postoperative pain in adults: a systematic review and meta-analysis of randomized trials. Can J Anaesth. (2016) 63:311–25. 10.1007/s12630-015-0551-4 26659198

[61] Assouline B Tramer MR Kreienbuhl L Elia N. Benefit and harm of adding ketamine to an opioid in a patient-controlled analgesia device for the control of postoperative pain: systematic review and meta-analyses of randomized controlled trials with trial sequential analyses. Pain. (2016) 157:2854–64. 10.1097/j.pain.0000000000000705 27780181

[62] Pendi A Field R Farhan SD Eichler M Bederman SS. Perioperative ketamine for analgesia in spine surgery: a meta-analysis of randomized controlled trials. Spine (Phila PA 1976). (2018) 43:E299–E307. 10.1097/BRS.0000000000002318 28700455 PMC5846492

[63] Bi Y Ye Y Zhu Y Ma J Zhang X Liu B. The effect of ketamine on acute and chronic wound pain in patients undergoing breast surgery: a meta-analysis and systematic review. Pain Pract. (2021) 21:316–32. 10.1111/papr.12961 33150677

[64] Kroin JS Das V Moric M Buvanendran A. Efficacy of the ketamine metabolite (2r,6r)-hydroxynorketamine in mice models of pain. Reg Anesth Pain Med. (2019) 44:111–7. 10.1136/rapm-2018-000013 30640662

[65] Swartjes M Morariu A Niesters M Aarts L Dahan A. Nonselective and Nr2b-selective N-methyl-D-aspartic acid receptor antagonists produce antinociception and long-term relief of Allodynia in acute and neuropathic pain. Anesthesiology. (2011) 115:165–74. 10.1097/ALN.0b013e31821bdb9b 21606828

[66] Velzen MV Dahan JDC van Dorp ELA Mogil JS Hooijmans CR Dahan A. Efficacy of ketamine in relieving neuropathic pain: a systematic review and meta-analysis of animal studies. Pain. (2021) 162:2320–30. 10.1097/j.pain.0000000000002231 33790195 PMC8374709

[67] Eichenberger U Neff F Sveticic G Bjorgo S Petersen-Felix S Arendt-Nielsen L. Chronic phantom limb pain: the effects of calcitonin, ketamine, and their combination on pain and sensory thresholds. Anesth Analg. (2008) 106:1265–73, table of contents. 10.1213/ane.0b013e3181685014 18349204

[68] Schwartzman RJ Alexander GM Grothusen JR Paylor T Reichenberger E Perreault M. Outpatient intravenous ketamine for the treatment of complex regional pain syndrome: a double-blind placebo controlled study. Pain. (2009) 147:107–15. 10.1016/j.pain.2009.08.015 19783371

[69] Mitchell AC Fallon MT. A single infusion of intravenous ketamine improves pain relief in patients with critical limb ischaemia: results of a double blind randomised controlled trial. Pain. (2002) 97:275–81. 10.1016/S0304-3959(02)00033-7 12044624

[70] Eide PK Stubhaug A Oye I Breivik H. continuous subcutaneous administration of the N-Methyl-D-aspartic acid (Nmda) receptor antagonist ketamine in the treatment of post-herpetic neuralgia. Pain. (1995) 61:221–8. 10.1016/0304-3959(94)00182-E 7659432

[71] Eide PK Jorum E Stubhaug A Bremnes J Breivik H. Relief of post-herpetic neuralgia with the N-Methyl-D-Aspartic acid receptor antagonist ketamine: a double-blind, cross-over comparison with morphine and placebo. Pain. (1994) 58:347–54. 10.1016/0304-3959(94)90129-5 7838584

[72] Nikolajsen L Hansen CL Nielsen J Keller J Arendt-Nielsen L Jensen TS. The effect of ketamine on phantom pain: a central neuropathic disorder maintained by peripheral input. Pain. (1996) 67:69–77. 10.1016/0304-3959(96)03080-1 8895233

[73] Dahan A Olofsen E Sigtermans M Noppers I Niesters M Aarts L. Population pharmacokinetic-pharmacodynamic modeling of ketamine-induced pain relief of chronic pain. Eur J Pain. (2011) 15:258–67. 10.1016/j.ejpain.2010.06.016 20638877

[74] Goldberg DS McGee SJ. Pain as a global public health priority. BMC Public Health. (2011) 11:770. 10.1186/1471-2458-11-770 21978149 PMC3201926

[75] Treede RD Rief W Barke A Aziz Q Bennett MI Benoliel R. Chronic pain as a symptom or a disease: the Iasp classification of chronic pain for the international classification of diseases (Icd-11). Pain. (2019) 160:19–27. 10.1097/j.pain.0000000000001384 30586067

[76] Macrae WA. Chronic post-surgical pain: 10 years on. Br J Anaesth. (2008) 101:77–86. 10.1093/bja/aen099 18434337

[77] Weiser TG Haynes AB Molina G Lipsitz SR Esquivel MM Uribe-Leitz T. Size and distribution of the global volume of surgery in 2012. Bull World Health Organ. (2016) 94:201–9F. 10.2471/BLT.15.159293 26966331 PMC4773932

[78] Baker DW. History of the joint commission's pain standards: lessons for today's prescription opioid epidemic. JAMA. (2017) 317:1117–8. 10.1001/jama.2017.0935 28241189

[79] WHO. International Classification of Diseases for Mortality and Morbidity Statistics. 11th ed. Geneva: World Health Orgnization (2019).

[80] Kuner R Flor H. Structural plasticity and reorganisation in chronic pain. Nat Rev Neurosci. (2016) 18:20–30. 10.1038/nrn.2016.162 27974843

[81] Peirs C Seal RP. Neural circuits for pain: recent advances and current views. Science. (2016) 354:578–84. 10.1126/science.aaf8933 27811268 PMC11327866

[82] Pozek JP Beausang D Baratta JL Viscusi ER. The acute to chronic pain transition: can chronic pain be prevented? Med Clin North Am. (2016) 100:17–30. 10.1016/j.mcna.2015.08.005 26614716

[83] Katz J Jackson M Kavanagh BP Sandler AN. Acute pain after thoracic surgery predicts long-term post-thoracotomy pain. Clin J Pain. (1996) 12:50–5. 10.1097/00002508-199603000-00009 8722735

[84] McCartney CJ Sinha A Katz J. A qualitative systematic review of the role of N-Methyl-D-aspartate receptor antagonists in preventive analgesia. Anesth Analg. (2004) 98:1385–400, table of contents. 10.1213/01.ANE.0000108501.57073.38 15105220

[85] Hirota K Lambert DG. Ketamine: new uses for an old drug? Br J Anaesth. (2011) 107:123–6. 10.1093/bja/aer221 21757548

[86] Lim J Chen D McNicol E Sharma L Varaday G Sharma A. Risk factors for persistent pain after breast and thoracic surgeries: a systematic literature review and meta-analysis. Pain. (2022) 163:3–20. 10.1097/j.pain.0000000000002301 34001769

[87] Jin J Chen Q Min S Du X Zhang D Qin P. Prevalence and predictors of chronic postsurgical pain after colorectal surgery: a prospective study. Colorectal Dis. (2021) 23:1878–89. 10.1111/codi.15640 33738887

[88] Gungor S Fields K Aiyer R Valle AGD Su EP. Incidence and risk factors for development of persistent postsurgical pain following total knee arthroplasty: a retrospective cohort study. Medicine (Baltimore). (2019) 98:e16450. 10.1097/MD.0000000000016450 31305475 PMC6641667

[89] Giusti EM Lacerenza M Gabrielli S Manzoni GM Manna C D'Amario F. Psychological factors and trajectories of post-surgical pain: a longitudinal prospective study. Pain Pract. (2021) 22:159–70. 10.1111/papr.13074 34498384

[90] Benlolo S Hanlon JG Shirreff L Lefebvre G Husslein H Shore EM. Predictors of persistent postsurgical pain after hysterectomy-a prospective cohort study. J Minim Invasive Gynecol. (2021) 28:2036–46 e1. 10.1016/j.jmig.2021.05.017 34077793

[91] Dindo L Zimmerman MB Hadlandsmyth K StMarie B Embree J Marchman J. Acceptance and commitment therapy for prevention of chronic postsurgical pain and opioid use in at-risk veterans: a pilot randomized controlled study. J Pain. (2018) 19:1211–21. 10.1016/j.jpain.2018.04.016 29777950 PMC6163061

[92] Nicholls JL Azam MA Burns LC Englesakis M Sutherland AM Weinrib AZ. Psychological treatments for the management of postsurgical pain: a systematic review of randomized controlled trials. Patient Relat Outcome Meas. (2018) 9:49–64. 10.2147/PROM.S121251 29403322 PMC5783145

[93] Zhang K Xu T Yuan Z Wei Z Yamaki VN Huang M. Essential roles of ampa receptor glua1 phosphorylation and presynaptic hcn channels in fast-acting antidepressant responses of ketamine. Sci Signal. (2016) 9:ra123. 10.1126/scisignal.aai7884 27965425 PMC5564288

[94] Zanos P Moaddel R Morris PJ Georgiou P Fischell J Elmer GI. Nmdar inhibition-independent antidepressant actions of ketamine metabolites. Nature. (2016) 533:481–6. 10.1038/nature17998 27144355 PMC4922311

[95] Yang C Yang J Luo A Hashimoto K. Molecular and cellular mechanisms underlying the antidepressant effects of ketamine enantiomers and its metabolites. Transl Psychiatry. (2019) 9:280. 10.1038/s41398-019-0624-1 31699965 PMC6838457

[96] Wei Y Chang L Hashimoto K. Molecular mechanisms underlying the antidepressant actions of arketamine: beyond the NMDA receptor. Mol Psychiatry. (2021) 27:559–73. 10.1038/s41380-021-01121-1 33963284 PMC8960399

[97] Cuttle MF Rusznak Z Wong AY Owens S Forsythe ID. Modulation of a presynaptic hyperpolarization-activated cationic current (I_h_) at an excitatory synaptic terminal in the rat auditory brainstem. J Physiol. (2001) 534(Pt 3):733–44. 10.1111/j.1469-7793.2001.00733.x 11483704 PMC2278738

[98] Luján R Albasanz JL Shigemoto R Juiz JM. Preferential localization of the hyperpolarization-activated cyclic nucleotide-gated cation channel subunit hcn1 in basket cell terminals of the rat cerebellum. Eur J Neurosci. (2005) 21:2073–82. 10.1111/j.1460-9568.2005.04043.x 15869503

[99] Boyes J Bolam JP Shigemoto R Stanford IM. Functional presynaptic Hcn channels in the rat globus pallidus. Eur J Neurosci. (2007) 25:2081–92. 10.1111/j.1460-9568.2007.05463.x 17439493

[100] Huang H Trussell LO. Presynaptic Hcn channels regulate vesicular glutamate transport. Neuron. (2014) 84:340–6. 10.1016/j.neuron.2014.08.046 25263752 PMC4254032

[101] Huang Z Li G Aguado C Lujan R Shah MM. Hcn1 channels reduce the rate of exocytosis from a subset of cortical synaptic terminals. Scientific Rep. (2017) 7:40257. 10.1038/srep40257 28071723 PMC5223132

[102] Huang Z Lujan R Kadurin I Uebele VN Renger JJ Dolphin AC. Presynaptic Hcn1 channels regulate Ca_v_3.2 activity and neurotransmission at select cortical synapses. Nat Neurosci. (2011) 14:478–86. 10.1038/nn.2757 21358644 PMC3068302

[103] Huang Z Lujan R Martinez-Hernandez J Lewis AS Chetkovich DM Shah MM. Trip8b-Independent trafficking and plasticity of adult cortical presynaptic Hcn1 channels. J Neurosci. (2012) 32:14835–48. 10.1523/JNEUROSCI.1544-12.2012 23077068 PMC4104472

[104] Mashour GA Ben Abdallah A Pryor KO El-Gabalawy R Vlisides PE Jacobsohn E. Intraoperative ketamine for prevention of depressive symptoms after major surgery in older adults: an international, multicentre, double-blind, randomised clinical trial. Br J Anaesth. (2018) 121:1075–83. 10.1016/j.bja.2018.03.030 30336852 PMC6208292

[105] Avidan MS Maybrier HR Abdallah AB Jacobsohn E Vlisides PE Pryor KO. Intraoperative ketamine for prevention of postoperative delirium or pain after major surgery in older adults: an international, multicentre, double-blind, randomised clinical trial. Lancet. (2017) 390:267–75. 10.1016/S0140-6736(17)31467-8 28576285 PMC5644286

[106] Kroenke K Bair MJ Damush TM Wu J Hoke S Sutherland J. Optimized antidepressant therapy and pain self-management in primary care patients with depression and musculoskeletal pain: a randomized controlled trial. JAMA. (2009) 301:2099–110. 10.1001/jama.2009.723 19470987 PMC2884224

[107] Park LT Luckenbaugh DA Pennybaker SJ Hopkins MA Henter ID Lener MS. The effects of ketamine on typical and atypical depressive symptoms. Acta Psychiatrica Scandinavica. (2020) 142:394–401. 10.1111/acps.13216 32677051 PMC10072788

[108] Vlisides PE Bel-Bahar T Nelson A Chilton K Smith E Janke E. Subanaesthetic ketamine and altered states of consciousness in humans. Br J Anaesth. (2018) 121:249–59. 10.1016/j.bja.2018.03.011 29935579 PMC6200112

[109] Van Essen DC. A population-average, landmark- and surface-based (Pals) atlas of human cerebral cortex. Neuroimage. (2005) 28:635–62. 10.1016/j.neuroimage.2005.06.058 16172003

[110] Geng JJ Vossel S. Re-Evaluating the role of Tpj in attentional control: contextual updating? Neurosci Biobehav Rev. (2013) 37(10 Pt 2):2608–20. 10.1016/j.neubiorev.2013.08.010 23999082 PMC3878596

[111] Bzdok D Heeger A Langner R Laird AR Fox PT Palomero-Gallagher N. Subspecialization in the human posterior medial cortex. Neuroimage. (2015) 106:55–71. 10.1016/j.neuroimage.2014.11.009 25462801 PMC4780672

[112] Braga RM Buckner RL. Parallel interdigitated distributed networks within the individual estimated by intrinsic functional connectivity. Neuron. (2017) 95:457–71 e5. 10.1016/j.neuron.2017.06.038 28728026 PMC5519493

[113] Krönke KM Wolff M Shi Y Kraplin A Smolka MN Bühringer G. Functional connectivity in a triple-network saliency model is associated with real-life self-control. Neuropsychologia. (2020) 149:107667. 10.1016/j.neuropsychologia.2020.107667 33130158

[114] Krall SC Rottschy C Oberwelland E Bzdok D Fox PT Eickhoff SB. The role of the right temporoparietal junction in attention and social interaction as revealed by ALE meta-analysis. Brain Struct Funct. (2015) 220:587–604. 10.1007/s00429-014-0803-z 24915964 PMC4791048

[115] Igelström KM Graziano MSA. The inferior parietal lobule and temporoparietal junction: a network perspective. Neuropsychologia. (2017) 105:70–83. 10.1016/j.neuropsychologia.2017.01.001 28057458

[116] Mars RB Sallet J Schuffelgen U Jbabdi S Toni I Rushworth MF. Connectivity-Based subdivisions of the human right temporoparietal junction area: evidence for different areas participating in different cortical networks. Cereb Cortex. (2012) 22:1894–903. 10.1093/cercor/bhr268 21955921

[117] Krall SC Volz LJ Oberwelland E Grefkes C Fink GR Konrad K. The right temporoparietal junction in attention and social interaction: a transcranial magnetic stimulation study. Human Brain Mapp. (2016) 37:796–807. 10.1002/hbm.23068 26610283 PMC6867405

[118] Blanke O. Multisensory brain mechanisms of bodily self-consciousness. Nat Rev Neurosci. (2012) 13:556–71. 10.1038/nrn3292 22805909

[119] Blanke O Ortigue S Landis T Seeck M. Stimulating illusory own-body perceptions. Nature. (2002) 419:269–70. 10.1038/419269a 12239558

[120] Blanke O Arzy S. The out-of-Body experience: disturbed self-processing at the temporo-parietal junction. Neuroscientist. (2005) 11:16–24. 10.1177/1073858404270885 15632275

[121] Wen X Liu Y Zhao P Liu Z Li H Li W. Disrupted communication of the temporoparietal junction in patients with major depressive disorder. Cogn Affect Behav Neurosci. (2021) 21:1276–96. 10.3758/s13415-021-00918-5 34100255

[122] McGowan JC LaGamma CT Lim SC Tsitsiklis M Neria Y Brachman RA. Prophylactic ketamine attenuates learned fear. Neuropsychopharmacology. (2017) 42:1577–89. 10.1038/npp.2017.19 28128336 PMC5518899

[123] Mathai DS Meyer MJ Storch EA Kosten TR. The relationship between subjective effects induced by a single dose of ketamine and treatment response in patients with major depressive disorder: a systematic review. J Affective Disorder. (2020) 264:123–9. 10.1016/j.jad.2019.12.023 32056741

[124] Sos P Klirova M Novak T Kohutova B Horacek J Palenicek T. Relationship of ketamine's antidepressant and psychotomimetic effects in unipolar depression. Neuro Endocrinol Lett. (2013) 34:287–93. 23803871

[125] Phillips JL Norris S Talbot J Birmingham M Hatchard T Ortiz A. Single, repeated, and maintenance ketamine infusions for treatment-resistant depression: a randomized controlled trial. Am J Psychiatry. (2019) 176:401–9. 10.1176/appi.ajp.2018.18070834 30922101

[126] Luckenbaugh DA Niciu MJ Ionescu DF Nolan NM Richards EM Brutsche NE. Do the dissociative side effects of ketamine mediate its antidepressant effects? J Affective Disorder. (2014) 159:56–61. 10.1016/j.jad.2014.02.017 24679390 PMC4065787

[127] Olofsen E Kamp J Henthorn TK van Velzen M Niesters M Sarton E. Ketamine psychedelic and antinociceptive effects are connected. Anesthesiology. (2022) 136:792–801. 10.1097/ALN.0000000000004176 35188952

[128] Vesuna S Kauvar IV Richman E Gore F Oskotsky T Sava-Segal C. Deep posteromedial cortical rhythm in dissociation. Nature. (2020) 586:87–94. 10.1038/s41586-020-2731-9 32939091 PMC7553818

[129] Vogt BA Paxinos G. Cytoarchitecture of mouse and rat cingulate cortex with human homologies. Brain Struct Funct. (2014) 219:185–92. 10.1007/s00429-012-0493-3 23229151

[130] Zilles K Eickhoff S Palomero-Gallagher N. The human parietal cortex: a novel approach to its architectonic mapping. Adv Neurol. (2003) 93:1–21. 12894398

[131] Cavanna AE Trimble MR. The precuneus: a review of its functional anatomy and behavioural correlates. Brain. (2006) 129(Pt 3):564–83. 10.1093/brain/awl004 16399806

[132] Cauda F Geminiani G D'Agata F Sacco K Duca S Bagshaw AP. Functional connectivity of the posteromedial cortex. PLoS ONE. (2010) 5:e13107. 10.1371/journal.pone.0013107 20927345 PMC2948030

[133] Broyd SJ Demanuele C Debener S Helps SK James CJ Sonuga-Barke EJ. Default-Mode brain dysfunction in mental disorders: a systematic review. Neurosci Biobehav Rev. (2009) 33:279–96. 10.1016/j.neubiorev.2008.09.002 18824195

[134] Parvizi J Braga RM Kucyi A Veit MJ Pinheiro-Chagas P Perry C. Altered sense of self during seizures in the posteromedial cortex. Proc Natl Acad Sci USA. (2021) 118:e2100522118. 10.1073/pnas.2100522118 34272280 PMC8307613

[135] American Psychiatric Association. Diagnostic and Statistical Manual of Mental Disorders. 5th ed. Arlington, VA (2013). 10.1176/appi.books.9780890425596

[136] Anand A Charney DS Oren DA Berman RM Hu XS Cappiello A. Attenuation of the neuropsychiatric effects of ketamine with lamotrigine: support for hyperglutamatergic effects of N-Methyl-D-aspartate receptor antagonists. Arch Gen Psychiatry. (2000) 57:270–6. 10.1001/archpsyc.57.3.270 10711913

[137] Sills GJ Rogawski MA. Mechanisms of action of currently used antiseizure drugs. Neuropharmacology. (2020) 168:107966. 10.1016/j.neuropharm.2020.107966 32120063

[138] Poolos NP Migliore M Johnston D. Pharmacological upregulation of H-channels reduces the excitability of pyramidal neuron dendrites. Nat Neurosci. (2002) 5:767–74. 10.1038/nn891 12118259

[139] Suarez LE Markello RD Betzel RF Misic B. Linking structure and function in macroscale brain networks. Trends Cogn Sci. (2020) 24:302–15. 10.1016/j.tics.2020.01.008 32160567

[140] Zhang J Kucyi A Raya J Nielsen AN Nomi JS Damoiseaux JS. What have we really learned from functional connectivity in clinical populations? Neuroimage. (2021) 242:118466. 10.1016/j.neuroimage.2021.118466 34389443

[141] Haber SN Liu H Seidlitz J Bullmore E. Prefrontal connectomics: from anatomy to human imaging. Neuropsychopharmacology. (2022) 47:20–40. 10.1038/s41386-021-01156-6 34584210 PMC8617085

[142] Menon V D'Esposito M. The role of Pfc networks in cognitive control and executive function. Neuropsychopharmacology. (2022) 47:90–103. 10.1038/s41386-021-01152-w 34408276 PMC8616903

[143] Power JD Cohen AL Nelson SM Wig GS Barnes KA Church JA. Functional network organization of the human brain. Neuron. (2011) 72:665–78. 10.1016/j.neuron.2011.09.006 22099467 PMC3222858

[144] Power JD Schlaggar BL Lessov-Schlaggar CN Petersen SE. Evidence for hubs in human functional brain networks. Neuron. (2013) 79:798–813. 10.1016/j.neuron.2013.07.035 23972601 PMC3838673

[145] Yeo BT Krienen FM Sepulcre J Sabuncu MR Lashkari D Hollinshead M. The organization of the human cerebral cortex estimated by intrinsic functional connectivity. J Neurophysiol. (2011) 106:1125–65. 10.1152/jn.00338.2011 21653723 PMC3174820

[146] Buckner RL Krienen FM Castellanos A Diaz JC Yeo BT. The organization of the human cerebellum estimated by intrinsic functional connectivity. J Neurophysiol. (2011) 106:2322–45. 10.1152/jn.00339.2011 21795627 PMC3214121

[147] Biswal B Yetkin FZ Haughton VM Hyde JS. Functional connectivity in the motor cortex of resting human brain using echo-planar Mri. Magnet Resonance Med. (1995) 34:537–41. 10.1002/mrm.1910340409 8524021

[148] Cordes D Haughton VM Arfanakis K Wendt GJ Turski PA Moritz CH. Mapping functionally related regions of brain with functional connectivity Mr imaging. AJNR American J Neuroradiol. (2000) 21:1636–44. 11039342 PMC8174861

[149] Lowe MJ Mock BJ Sorenson JA. Functional connectivity in single and multislice echoplanar imaging using resting-state fluctuations. Neuroimage. (1998) 7:119–32. 10.1006/nimg.1997.0315 9558644

[150] Dosenbach NU Fair DA Miezin FM Cohen AL Wenger KK Dosenbach RA. Distinct brain networks for adaptive and stable task control in humans. Proc Natl Acad Sci USA. (2007) 104:11073–8. 10.1073/pnas.0704320104 17576922 PMC1904171

[151] Gordon EM Lynch CJ Gratton C Laumann TO Gilmore AW Greene DJ. Three distinct sets of connector hubs integrate human brain function. Cell Rep. (2018) 24:1687–95 e4. 10.1016/j.celrep.2018.07.050 30110625 PMC6886580

[152] Greicius MD Supekar K Menon V Dougherty RF. Resting-state functional connectivity reflects structural connectivity in the default mode network. Cereb Cortex. (2009) 19:72–8. 10.1093/cercor/bhn059 18403396 PMC2605172

[153] Buckner RL Andrews-Hanna JR Schacter DL. The brain's default network: anatomy, function, and relevance to disease. Ann N Y Acad Sci. (2008) 1124:1–38. 10.1196/annals.1440.011 18400922

[154] Del Fabro L Schmidt A Fortea L Delvecchio G D'Agostino A Radua J. Functional brain network dysfunctions in subjects at high-risk for psychosis: a meta-analysis of resting-state functional connectivity. Neurosci Biobehav Rev. (2021) 128:90–101. 10.1016/j.neubiorev.2021.06.020 34119524

[155] Hu ML Zong XF Mann JJ Zheng JJ Liao YH Li ZC. A review of the functional and anatomical default mode network in schizophrenia. Neurosci Bull. (2017) 33:73–84. 10.1007/s12264-016-0090-1 27995564 PMC5567552

[156] Kircher T Brohl H Meier F Engelen J. Formal thought disorders: from phenomenology to neurobiology. Lancet Psychiatry. (2018) 5:515–26. 10.1016/S2215-0366(18)30059-2 29678679

[157] Song M Zhang Y Cui Y Yang Y Jiang T. Brain network studies in chronic disorders of consciousness: advances and perspectives. Neurosci Bull. (2018) 34:592–604. 10.1007/s12264-018-0243-5 29916113 PMC6060221

[158] Malaia E Bates E Seitzman B Coppess K. Altered brain network dynamics in youths with autism spectrum disorder. Exp Brain Res. (2016) 234:3425–31. 10.1007/s00221-016-4737-y 27465558 PMC5097108

[159] Scheidegger M Walter M Lehmann M Metzger C Grimm S Boeker H. Ketamine decreases resting state functional network connectivity in healthy subjects: implications for antidepressant drug action. PLoS ONE. (2012) 7:e44799. 10.1371/journal.pone.0044799 23049758 PMC3461985

[160] Bonhomme V Vanhaudenhuyse A Demertzi A Bruno MA Jaquet O Bahri MA. Resting-state network-specific breakdown of functional connectivity during ketamine alteration of consciousness in volunteers. Anesthesiology. (2016) 125:873–88. 10.1097/ALN.0000000000001275 27496657

[161] Mueller F Musso F London M de Boer P Zacharias N Winterer G. Pharmacological Fmri: effects of subanesthetic ketamine on resting-state functional connectivity in the default mode network, salience network, dorsal attention network and executive control network. Neuroimage Clin. (2018) 19:745–57. 10.1016/j.nicl.2018.05.037 30003027 PMC6040604

[162] Norbury A Rutter SB Collins AB Costi S Jha MK Horn SR. Neuroimaging correlates and predictors of response to repeated-dose intravenous ketamine in Ptsd: preliminary evidence. Neuropsychopharmacology. (2021) 46:2266–77. 10.1038/s41386-021-01104-4 34333555 PMC8580962

[163] Robinson OJ Pike AC Cornwell B Grillon C. The translational neural circuitry of anxiety. J Neurol Neurosurg Psychiatry. (2019) 90:1353–60. 10.1136/jnnp-2019-321400 31256001

[164] Murrough JW Collins KA Fields J DeWilde KE Phillips ML Mathew SJ. Regulation of neural responses to emotion perception by ketamine in individuals with treatment-resistant major depressive disorder. Transl Psychiatry. (2015) 5:e509. 10.1038/tp.2015.10 25689570 PMC4445748

[165] Reed JL Nugent AC Furey ML Szczepanik JE Evans JW Zarate CA Jr. Effects of ketamine on brain activity during emotional processing: differential findings in depressed versus healthy control participants. Biol Psychiatry Cogn Neurosci Neuroimaging. (2019) 4:610–8. 10.1016/j.bpsc.2019.01.005 30826253 PMC6612456

[166] Loureiro JRA Leaver A Vasavada M Sahib AK Kubicki A Joshi S. Modulation of amygdala reactivity following rapidly acting interventions for major depression. Human Brain Mapp. (2020) 41:1699–710. 10.1002/hbm.24895 32115848 PMC7268016

[167] Vasavada MM Loureiro J Kubicki A Sahib A Wade B Hellemann G. Effects of serial ketamine infusions on corticolimbic functional connectivity in major depression. Biol Psychiatry Cogn Neurosci Neuroimaging. (2021) 6:735–44. 10.1016/j.bpsc.2020.06.015 32900657 PMC8629108

[168] Kaiser RH Andrews-Hanna JR Wager TD Pizzagalli DA. Large-scale network dysfunction in major depressive disorder: a meta-analysis of resting-state functional connectivity. JAMA Psychiatry. (2015) 72:603–11. 10.1001/jamapsychiatry.2015.0071 25785575 PMC4456260

[169] Gärtner M Aust S Bajbouj M Fan Y Wingenfeld K Otte C. Functional connectivity between prefrontal cortex and subgenual cingulate predicts antidepressant effects of ketamine. Eur Neuropsychopharmacol. (2019) 29:501–8. 10.1016/j.euroneuro.2019.02.008 30819549

[170] Wilkinson ST Holtzheimer PE Gao S Kirwin DS Price RB. Leveraging neuroplasticity to enhance adaptive learning: the potential for synergistic somatic-behavioral treatment combinations to improve clinical outcomes in depression. Biol Psychiatry. (2019) 85:454–65. 10.1016/j.biopsych.2018.09.004 30528745 PMC6380941

[171] Hillman EM. Coupling mechanism and significance of the bold signal: a status report. Annu Rev Neurosci. (2014) 37:161–81. 10.1146/annurev-neuro-071013-014111 25032494 PMC4147398

[172] Purdon PL Sampson A Pavone KJ Brown EN. Clinical electroencephalography for anesthesiologists: part i: background and basic signatures. Anesthesiology. (2015) 123:937–60. 10.1097/ALN.0000000000000841 26275092 PMC4573341

[173] Buzsáki G. Rhythms of the Brain. New York: Oxford University Press. (2006).

[174] Cebolla AM Cheron G. Understanding neural oscillations in the human brain: from movement to consciousness and vice versa. FrontPsychol. (2019) 10:1930. 10.3389/fpsyg.2019.01930 31507490 PMC6718699

[175] Buzsáki G Watson BO. Brain rhythms and neural syntax: implications for efficient coding of cognitive content and neuropsychiatric disease. Dialogues Clin Neurosci. (2012) 14:345–67. 10.31887/DCNS.2012.14.4/gbuzsaki 23393413 PMC3553572

[176] Brookes MJ Tewarie PK Hunt BAE Robson SE Gascoyne LE Liddle EB. A multi-layer network approach to meg connectivity analysis. Neuroimage. (2016) 132:425–38. 10.1016/j.neuroimage.2016.02.045 26908313 PMC4862958

[177] Nugent AC Ballard ED Gilbert JR Tewarie PK Brookes MJ Zarate CA Jr. The effect of ketamine on electrophysiological connectivity in major depressive disorder. Front Psychiatry. (2020) 11:519. 10.3389/fpsyt.2020.00519 32655423 PMC7325927

[178] Bosma RL Cheng JC Rogachov A Kim JA Hemington KS Osborne NR. Brain dynamics and temporal summation of pain predicts neuropathic pain relief from ketamine infusion. Anesthesiology. (2018) 129:1015–24. 10.1097/ALN.0000000000002417 30199420

[179] Kucyi A Moayedi M Weissman-Fogel I Goldberg MB Freeman BV Tenenbaum HC. Enhanced medial prefrontal-default mode network functional connectivity in chronic pain and its association with pain rumination. J Neurosci. (2014) 34:3969–75. 10.1523/JNEUROSCI.5055-13.2014 24623774 PMC6705280

[180] Cheng JC Erpelding N Kucyi A DeSouza DD Davis KD. Individual differences in temporal summation of pain reflect pronociceptive and antinociceptive brain structure and function. J Neurosci. (2015) 35:9689–700. 10.1523/JNEUROSCI.5039-14.2015 26134651 PMC6605146

[181] Kucyi A Salomons TV Davis KD. Mind wandering away from pain dynamically engages antinociceptive and default mode brain networks. Proc Natl Acad Sci USA. (2013) 110:18692–7. 10.1073/pnas.1312902110 24167282 PMC3832014

[182] Motoyama Y Oshiro Y Takao Y Sato H Obata N Izuta S. Resting-state brain functional connectivity in patients with chronic pain who responded to subanesthetic-dose ketamine. Sci Rep. (2019) 9:12912. 10.1038/s41598-019-49360-1 31501482 PMC6733873

[183] Kucyi A Davis KD. The dynamic pain connectome. Trends Neurosci. (2015) 38:86–95. 10.1016/j.tins.2014.11.006 25541287

[184] Rogachov A Bhatia A Cheng JC Bosma RL Kim JA Osborne NR. Plasticity in the dynamic pain connectome associated with ketamine-induced neuropathic pain relief. Pain. (2019) 160:1670–9. 10.1097/j.pain.0000000000001545 30839433

[185] McNicol ED Schumann R Haroutounian S. A systematic review and meta-analysis of ketamine for the prevention of persistent post-surgical pain. Acta Anaesthesiol Scand. (2014) 58:1199–213. 10.1111/aas.12377 25060512

[186] Klatt E Zumbrunn T Bandschapp O Girard T Ruppen W. Intra- and postoperative intravenous ketamine does not prevent chronic pain: a systematic review and meta-analysis. Scand J Pain. (2015) 7:42–54. 10.1016/j.sjpain.2014.12.005 29911604

